# DNA Condensation-Inspired Assembly of DNA Nanotubes into Reversible Superstructures: A Base Pairing-Orthogonal Way to Create Rings, Bundles, or Vast Networks

**DOI:** 10.1021/jacs.5c10921

**Published:** 2025-10-01

**Authors:** Laura Bourdon, Xiang Zhen Xu, Laurent J. Michot, Mathieu Morel, Sergii Rudiuk, Ayako Yamada, Damien Baigl

**Affiliations:** † CPCV, Department of Chemistry, École Normale Supérieure, 26909 PSL University, Sorbonne Université, CNRS, Paris 75005, France; ‡ 52883 Laboratoire de Physique et d’Etude des Matériaux (LPEM), CNRS UMR 8213, ESPCI-Paris, PSL Research University, Sorbonne Université, 10 rue Vauquelin, Paris 75005 France; § 129925 Laboratory of Physical Chemistry of Electrolytes and Interfacial Nanosystems (PHENIX), UMR 8234 CNRS, Sorbonne University, Paris 75005, France

## Abstract

By offering exquisite programmability, sequence-specific DNA self-assembly is the foundation of structural DNA nanotechnology but necessitates custom-designed DNA strands. Finding assembly principles orthogonal to base pairing is thus desirable not only to organize DNA in a sequence-independent manner but also to bring additional levels of control over preformed DNA self-assembled structures. Here, we report that self-assembled DNA nanotubes, upon the addition of DNA-condensing multivalent cations, including the naturally occurring polyamines spermidine and spermine, spontaneously condense to form higher-order structures including well-defined micrometer-sized rings and 30 to 60 nm wide bundles, in which DNA strands are parallelly packed with an interspacing ranging from 2.5 to 3 nm. In the semidilute regime, a new organization into vast tridimensional networks is observed for a specific range of charge ratios, prior to the formation of highly clustered bundles. We demonstrate that the process is electrostatically driven, conferring a ubiquitous character to this assembly principle. We report in particular a pivotal role of the counterion valency (the higher it is, the lower the charge ratio required), emphasizing the role of DNA neutralization through the entropically driven exchange between DNA counterions and the condensing agents. We also show an important role of DNA concentration for controlling the individual or interconnected nature of the formed structures as well as favoring the nanotube assembly. We finally devise methods for additional control, such as superstructure disassembly upon monovalent ion addition or photocontrol using a photosensitive DNA-condensing agent.

## Graphical abstract

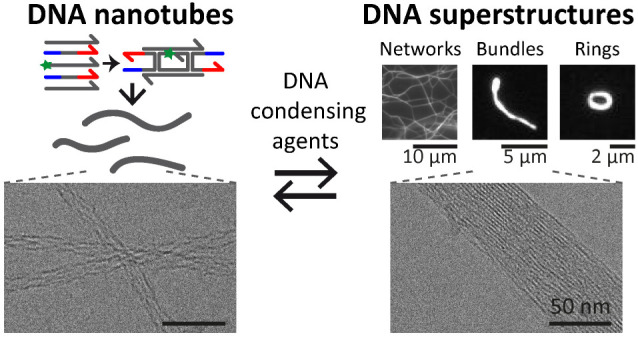

## Introduction

Structural DNA nanotechnology has emerged as a versatile tool to build elaborate nanostructures in a convenient, biocompatible, and user-friendly way.^1^ By exploiting specific DNA base-pairing principles, it is now possible to program the assembly of cocktails of synthetic DNA strands into virtually any desired 2D^2,3^ or 3D^4–6^ morphologies. The resulting structures are not only obtained at a high yield with great precision, but they can also be used as universal scaffolds to spatially organize bound entities (proteins, particles, etc.) with subnanometric resolution, leading to a vast range of applications from materials science to biomedicine.^7–9^ The underlying DNA self-assembly principles impose, however, some limits. First, most approaches rely on the use of a scaffold, as in the case of DNA origamis,^2^ which strongly limits the size of the final self-assembled structures, typically up to around 100 nm, unless specific additional protocols are applied.^10,11^ Additionally, despite the recent development of isothermal DNA self-assembly principles,^12^ the majority of methods rely on a thermal annealing step to ensure flawless assembly between the multitude of DNA strands.^2,13,14^ This temperature treatment, consisting of heating the system above the melting temperature before a slow cooling ramp, usually takes hours to days, thus imposing strong temporal constraints on the assembly. To expand the potential offered by programmable DNA self-assembly, it would be highly valuable to identify principles orthogonal to base-pairing rules in which DNA nanostructures could be assembled into well-defined superstructures, both extended in space and produced in a rapid manner. We suggest approaching such a goal by getting inspired by the fact that, in nature, DNA is usually present in a variety of higher-order structures not solely relying on Watson–Crick–Franklin interactions. For instance, genomic DNA of viruses, eukaryotes, and prokaryotes is highly organized thanks to a combination of interactions where electrostatics plays a major role. It has been shown in particular that double-stranded DNA, which adopts an elongated coil conformation in water due to the strong electrostatic repulsions between the phosphate groups along its backbone, undergoes a dramatic transition into highly ordered structures, such as toroids, when multivalent cations, including naturally occurring polyamines such as spermidine and spermine, are added.^15–17^

We envisaged that this principle could be used to rapidly reorganize self-assembled DNA nanostructures into higher-order superstructures. The exploitation of electrostatic interactions for such a purpose has already been explored but only in a few notable cases. In the case of DNA origamis, solid substrates were used to generate electrostatically tunable lattices^18–20^ or induce intramolecular suprafolding transitions in the case of soft cationic polymer multilayers.^21^ The requirement of solid substrates limits, however, the versatility and applicability of the resulting assemblies. In bulk, intramolecular reconfiguration upon the addition of positively charged proteins^22^ and intermolecular assemblies mediated by cationic nanoparticles^23^ were reported. However, DNA origamis, remaining small building blocks, are not well suited for the construction of extended 3D assemblies. For their elongated geometry and the possibility to reach micrometric dimensions, DNA nanotubes obtained by the self-assembly of single-stranded or double-crossover tiles appear as more promising starting materials.^24–28^ For instance, the addition of crowding agents or magnesium ions induced the formation of asters^29^ or bundles,^30–32^ respectively, while specifically designed macromolecular star-shaped cationic cross-linking agents led to contractile rings.^33^ Surprisingly, the use of naturally occurring polyamines for the assembly of DNA nanotubes has not been explored. Moreover, although DNA condensation is a reversible process, the possibility of dynamically disassembling DNA nanotube superstructures has been overlooked. Here, we used a simple cocktail of 5 DNA strands leading to long self-assembled nanotubes^24^ and studied how they reorganized upon the addition of two naturally occurring polyamines known as DNA condensing agents, the triamine spermidine (noted SPD^3+^) and the tetraamine spermine (noted SPM^4+^), and compared them with the effects of magnesium ions (Mg^2+^). Using fluorescence and electron microscopy as well as small-angle X-ray scattering (SAXS), we revealed the reproducible formation of a diversity of superstructures, including bundles, rings, and novel organizations into extended networks. We established phase diagrams highlighting the importance of counterion valency and DNA concentration. We finally established methods to reversibly disassemble the superstructures into nanotubes through monovalent salt addition and explored the possibility of photocontrolling the superstructure formation in the presence of a photosensitive DNA condensing agent.

## Results and Discussion

### Formation of Networks, Bundles, and Rings by Addition of the Tetravalent DNA Condensing Agent Spermine

We used DNA nanotubes obtained by the thermal annealing of 5 short DNA strands (500 nM each, including a fluorescently labeled one) in a so-called TAMg buffer (Trizma base 40 mM, acetic acid 20 mM, MgCl_2_ 12.5 mM). We hypothesized that the addition of multivalent cations capable of DNA condensation would induce the formation of superstructures that could be disassembled with the further addition of monovalent ions in a sufficiently large excess (Figure 1A). Initial nanotubes appeared as elongated filaments freely fluctuating in solution as observed by fluorescence microscopy (Figure 1B *left*, *top image*, and Movie S1). Transmission electron microscopy (TEM) revealed that they were well individualized (Figure 1B *left*, *bottom image*) with a uniform diameter of 12 ± 3 nm (Figure S1A), in agreement with previous reports.^24^ We first added spermine (SPM^4+^), a natural tetraamine, and observed the resulting structures in bulk by fluorescence microscopy. The addition of millimolar amounts of SPM^4+^ led to the immediate formation of arrested structures of large dimensions floating in solution, with a low fluorescence background indicating that most nanotubes were engaged in these clustered structures (Figure 1B *right*, *top image*, and Movie S1). Moreover, TEM revealed a local organization into thick elongated structures (Figure 1B *right*, *bottom image*) with an average diameter of 63 ± 26 nm (Figure S1B) where individual nanotubes could not be distinguished anymore, indicating a very strong attraction mediated by SPM^4+^. Similar assemblies were observed in the past using high concentrations of Mg^2+^ or crowding agents^30,32,33^ and will be referred to as “bundles.”

**Figure 1:**
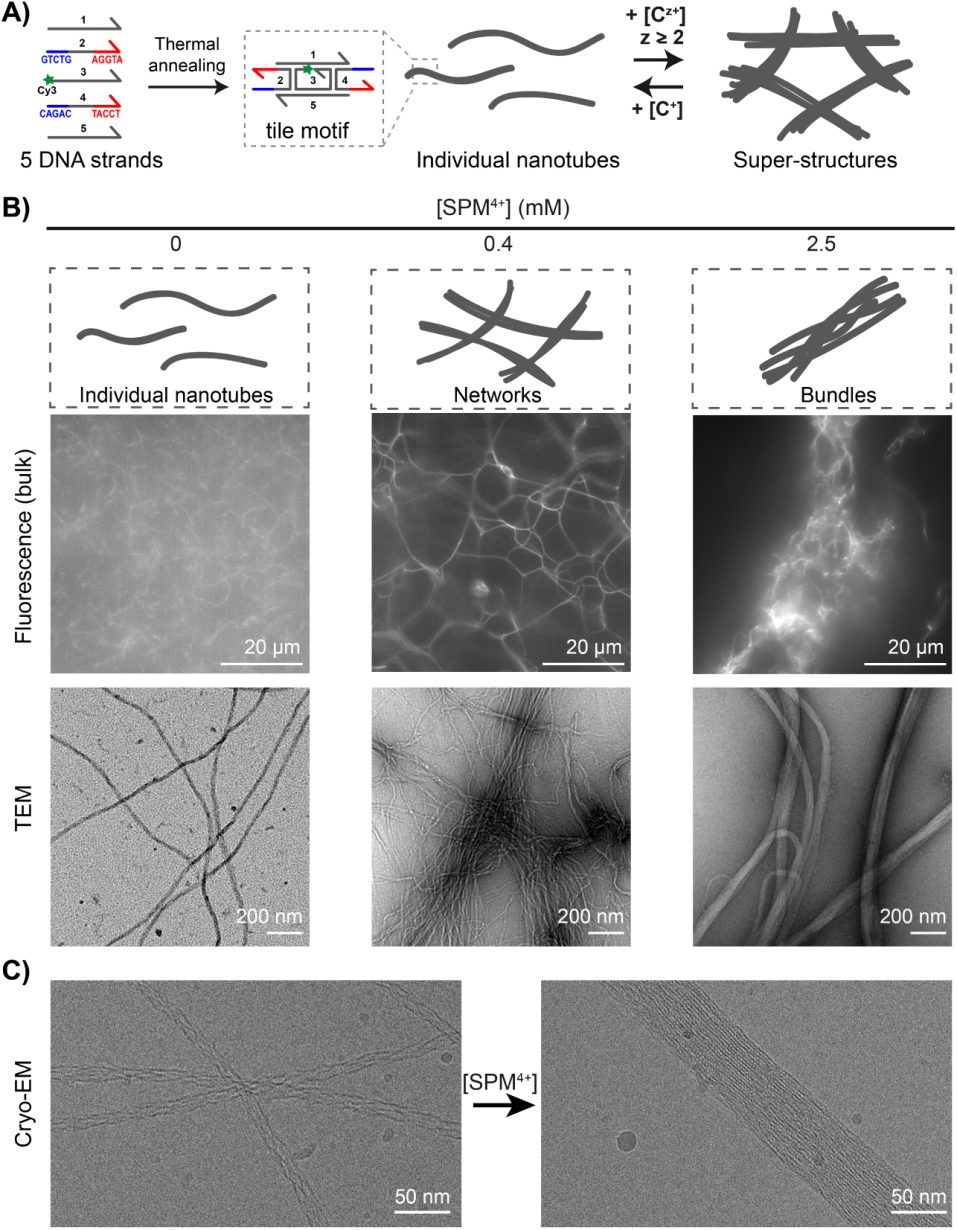
Multivalent cationic DNA-condensing agents induce the assembly of individual DNA nanotubes into various superstructures. A) Concept and experimental principle: individual nanotubes, prepared by the thermal assembly of five DNA strands forming a self-associating tile motif, assemble into superstructures upon the addition of multivalent cations (of valency *z*) that can be reversibly dissociated with an excess of monovalent cations. The central strand of the motif is labeled with Cy3 dye at its 5′ end. B) Characterization of individual nanotubes and superstructures obtained with 0, 0.4, and 2.5 mM of spermine (SPM^4+^), by epifluorescence microscopy (Cy3 fluorescence, top) and transmission electron microscopy (TEM, bottom). C) Cryo-electron microscopy of nanotubes before (left) and after (right) the addition of 2.5 mM of spermine. Each DNA strand concentration is 500 nM in TAMg buffer.

Notably, for intermediate SPM^4+^ concentration, we observed an original interconnected organization (Figure 1B *middle*). It consisted of highly fluorescent filaments connected into vast three-dimensional networks with a mesh size varying within the range of 5–20 μm (Figure 1B *middle*, *top image,* and Movie S1). Contrary to finite-size bundles, these interconnected structures occupied most of the solution. Interestingly, TEM revealed that individual nanotubes could be distinguished and were found to locally organize into aligned assemblies (Figure 1B *middle*, *bottom image*), showing intertube attraction, but without strongly condensing as in the bundles.

It is known that the electrostatic condensation of a semiflexible polyelectrolyte like double-stranded DNA (dsDNA) leads to the formation of toroids with a diameter (≈100 nm) around twice its persistence length (50 nm),^17^ inside which dsDNA double helices are parallelly packed in a nearly crystalline manner with an interspacing of about 2.4 nm.^34^ We thus scrutinized in more detail the regime of bundle formation ([SPM^4+^] = 2.5 mM). First, we used cryo-electron microscopy (cryo-EM) to reveal the internal structure of the DNA assemblies before and after bundle formation (Figure 1C). Without SPM^4+^, we could distinguish the DNA strands in the repeating tile motif forming hollow nanotubes with a diameter (12 ± 2 nm, *n* = 87) in agreement with TEM observations. Notably, after SPM^4+^ addition, hollow nanotubes could not be distinguished anymore. Instead, the inner structure of the bundles revealed a tightly packed parallel DNA arrangement with an interspacing of around 3 nm, i.e., a value close to that of a double-helix diameter (2 nm). This feature, structurally reminiscent of the internal organization of condensed DNA in toroids, confirms the role of SPM^4+^ to induce the electrostatic condensation of the nanotubes. Moreover, using TEM we detected that the condensed nanotubes also existed in the form of self-closed structures similar to DNA toroids but of much larger dimensions (Figure 2A,B). These rings were also observed by confocal microscopy coexisting with bundle fragments adsorbed on the microscopy coverslip surface (Figure 2C *left*, *white arrows*). Using super-resolution fluorescence imaging allowing a 120 nm lateral resolution, rings appeared with a single dense contour (Figure 2C *right*), in agreement with cryo-EM (Figure 1C *right*) and TEM (Figure 2B) data, confirming the compact longitudinal assemblies of the nanotubes in these structures. The perimeter-equivalent diameter of the rings was found to typically vary between 1 and 10 μm (Figure 2D), with a median of 1.3 μm and a mean ± SD value of 2.2 ± 1.9 μm (Figure 2E), which could indicate that the persistence length of condensed nanotubes became smaller than that of free nanotubes (≈4 μm)^24^ due to the local structural change evidenced by cryo-EM (Figure 1C). It is also in agreement with a recent report using a large synthetic star-shaped multicationic cross-linking agent.^33^ Our results thus show that electrostatic DNA condensation by counterion condensation is enough to generate the spontaneous formation of rings and, notably, does not require specific cross-linking interactions.

**Figure 2:**
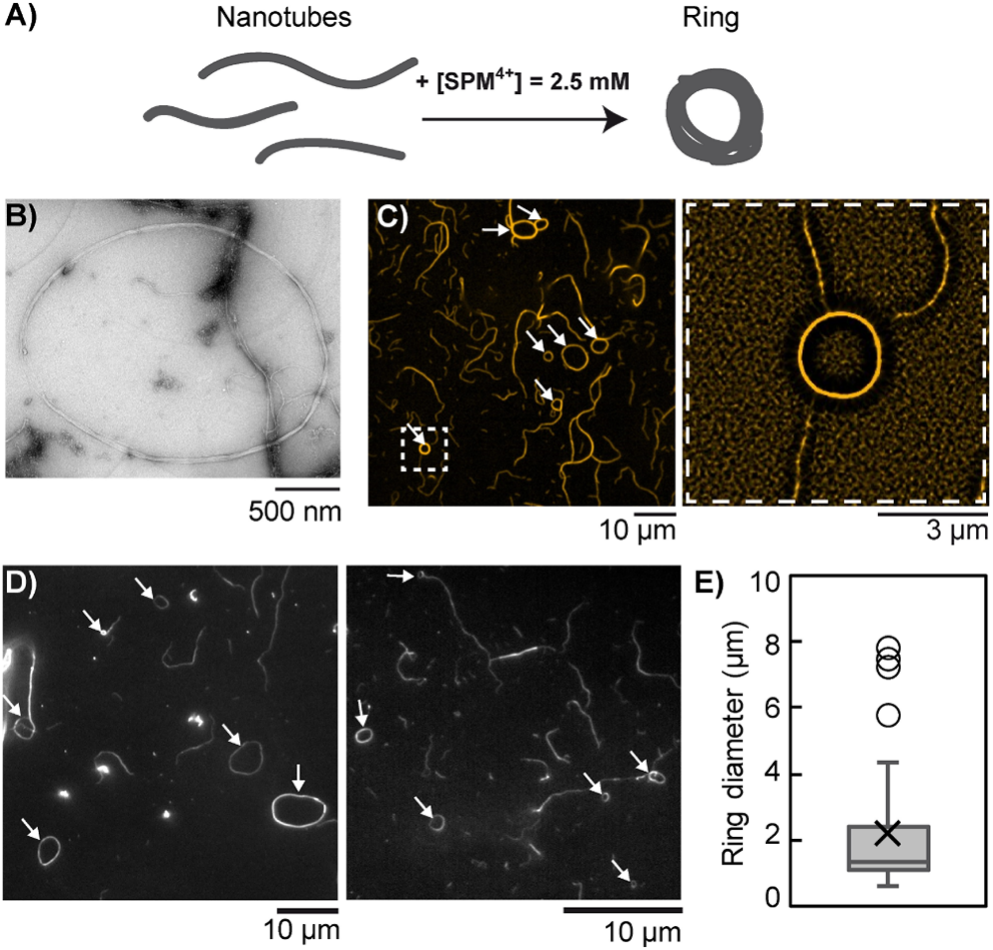
Sufficient spermine concentration (2.5 mM) induces the condensation of individual DNA nanotubes into ring-like structures. A) Scheme of ring formation. B) Representative TEM image of a ring. C) Left, confocal image showing the rings adsorbed (indicated by white arrows) on the surface of a coverslip. Right, super-resolved image of the ring boxed by a dashed line on the left. D) Epifluorescence microscopy images of DNA rings adsorbed on a glass coverslip. E) Box and whiskers plot (outliers included) of the ring diameter distribution established from epifluorescence imaging (*n* = 46). The box ranges from the first to the third quartile, with the median and average indicated with horizontal lines and a cross, respectively. Each DNA strand concentration is 500 nM in TAMg buffer.

### Effect of Condensing Agent Valency

To both understand the physicochemical mechanisms underlying the formation of the different superstructures and analyze how general this behavior could be, we established a diagram reporting the nature of the obtained superstructures for different multivalent cations added to DNA nanotubes. The diagram was plotted as a function of both the condensing agent concentration (Figure S2) and the charge ratio ρ (Figure 3A), which was defined as the concentration of charges brought by the condensing agent normalized by that of DNA (Text S1). Starting with spermine (SPM^4+^), we observed the formation of networks from [SPM^4+^] = 0.2 mM (ρ = 7.14) to 0.4 mM (ρ = 14.3) (Figure S3). For larger concentrations, nanotubes were organized into bundles coexisting with a fraction of rings, in agreement with Figures 1 and 2. Using spermidine (SPD^3+^), a trivalent polyamine, a similar nanotube-network transition was observed but at a higher concentration (5 mM, Figures S2 and S4). Interestingly, this concentration increase was larger than a simple compensation of valency, as revealed by the larger charge ratio at the transition (ρ = 143, Figure 3A). We compared the effect of these two polyamines to the simple dication Mg^2+^ and found the same behavior, at even a larger concentration (50 mM, Figures S2 and S5) and ρ (ρ = 1190, Figures 3A and S5), confirming not only the importance of valency in driving the process but also that such nanotube assembly mainly relies on electrostatic attraction rather than specific chemical interactions. For SPD^3+^, bundles were observed for higher concentrations and charge ratios (Figures 3A and S4) but were smaller in size (32 ± 9 nm, Figure S6) than with SPM^4+^, while we could not detect bundles in the highest magnesium concentrations tested in our experiments. This further highlights the importance of a high condensation agent valency to obtain large and dense assemblies of DNA nanotubes.

**Figure 3:**
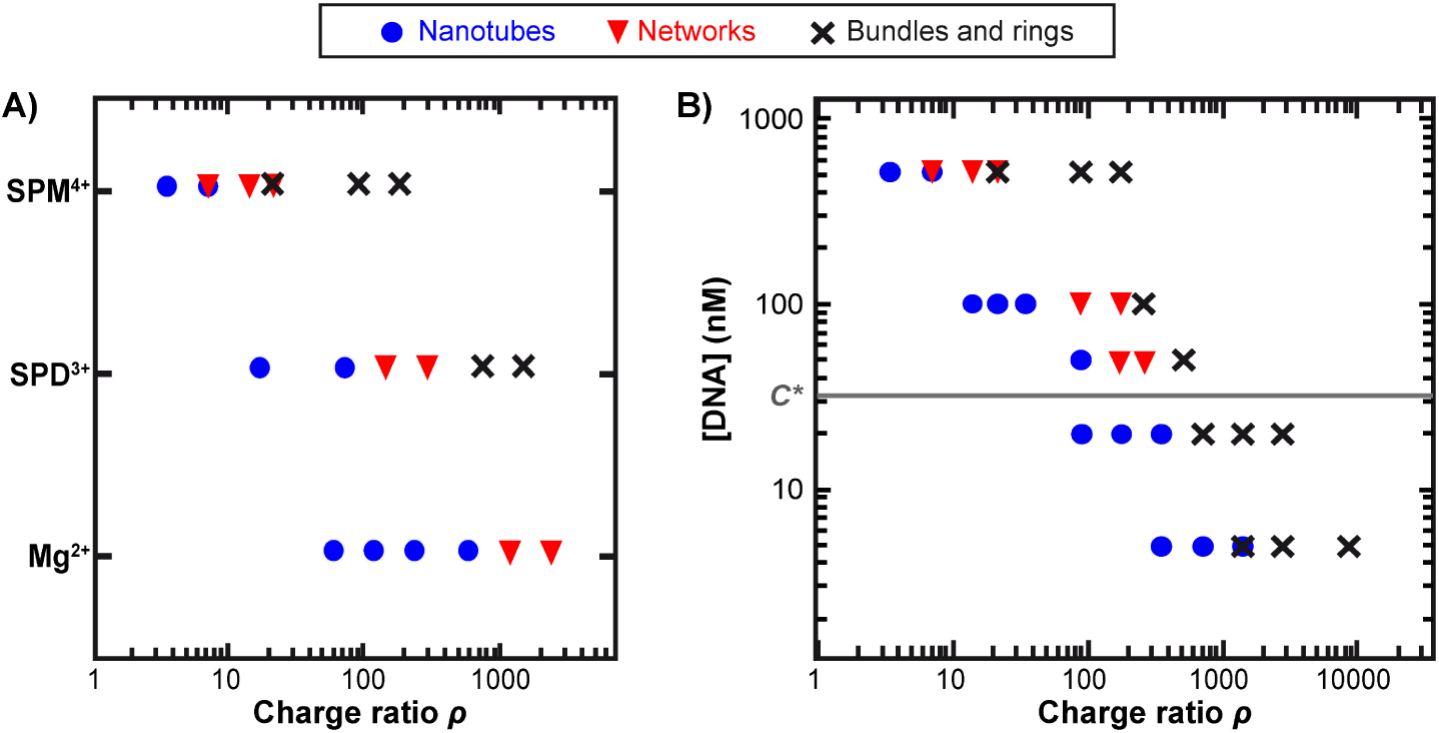
Nanotube assembly is controlled by the condensing agent valency and DNA concentration. A) Diagram showing the superstructures formed by the nanotubes after the addition of different multivalent cations (magnesium Mg^2+^, spermidine SPD^3+,^ or spermine SPM^4+^), as a function of the charge ratio ρ defined as the concentration of charges brought by the condensing agent normalized by that of DNA. Each DNA strand concentration is 500 nM in TAMg buffer. B) Diagram showing the superstructures formed by the nanotubes after the addition of spermine as a function of the charge ratio ρ for different DNA strand concentrations in TAMg buffer. The gray line represents the concentration corresponding to the transition between the dilute and the semidilute regime, estimated to be C* = 32 nM for a number of tiles per nanotube crosss-section n_tile_ = 7.

For a given valency, the evolution observed in all these phase diagrams is in agreement with the assembly of rigid negatively charged polyelectrolytes, such as actin filaments or microtubules, forming bundles in the presence of sufficiently high concentrations of multivalent cations, as shown in the past both experimentally and theoretically.^35–38^ The valency dependence is also reminiscent to the compaction of double-stranded DNA by multivalent cations, where the neutralization of the DNA backbone through counterion condensation drives the process.^39^ According to the Manning–Oosawa picture,^40,41^ counterions of valency *z* condense on the DNA backbone leading to an average charge neutralization θ = 1 – *d*/(*zl*_B_) where *d* is the average distance between DNA charges (0.17 nm) and *l*_B_ is the Bjerrum length (*l*_B_ = *e* ^2^/(4π*εk*_B_ *T*), with *e* the elementary charge, *ε* the dielectric constant of the solvent, *k*_B_ the Boltzmann constant, and *T* the temperature). In water at 25 °C, *l*_B_ = 0.7 nm and the neutralization only depends on *z*: θ = 1 – 0.24/*z*. Therefore, adding multivalent cations to double-stranded DNA induces an entropically favorable counterion exchange and a progressive neutralization of DNA leading to its compaction when θ becomes too large (≈0.89).^42^ The higher the valency, the more entropically favorable the counterion exchange is, the more efficient DNA neutralization becomes, and the lower is the charge ratio necessary to induce DNA compaction. Notably, the same evolution was observed in the nanotube condensation diagram, where transitions to higher-order structures were systematically observed at a lower ρ when *z* increased (Figure 3A).

All of these results showed the importance of cation valency to drive the formation of superstructures. Knowing that our buffer contained a significant amount of Mg^2+^ (12.5 mM), we performed the same study in a buffer composed of solely monovalent cations. To get stable nanotubes, we replaced MgCl_2_ by a high concentration of NaCl (100 mM).^12^ Interestingly, we obtained the same types of superstructures by adding increasing amounts of SPM^4+^ (Figure S7), including the formation of rings (Figure S8) having dimensions similar to those obtained in the regular Mg^2+^-containing buffer (Figure 2E). All these results showed that the multivalent counterions were the main driving force for the superstructure formation. Note that the frontiers for the transitions were shifted to higher charge ratios ρ when Na^+^ was used (ρ = 21.4 for the nanotube-network transition and ρ = 89.3 for the network-bundle transition, Figure S7) instead of Mg^2+^ (*ρ =* 7.14 and ρ = 14.3, respectively, Figure S3), attributed to the large concentration of monovalent cations competing for counterion exchange and DNA neutralization.

### Small Angle X-ray Scattering (SAXS) Study of Nanotube Assembly

To get an ensemble vision as well as further insights on the nanotube assembly in bulk upon the addition of DNA-condensing multivalent cations, we analyzed the SAXS curves of nanotubes at the DNA concentration used in the previous microscopy studies (500 nM per strand in TAMg buffer) for numerous spermine and spermidine concentrations (Figures S9 and S10). DNA nanotubes in these conditions were low scatterers, resulting in overall noisy curves yet with distinctive properties that could be extracted. Starting with spermine, we could distinguish in particular three characteristic regimes depending on spermine concentration (Figure S9). In Regime 1, corresponding to low spermine concentrations ranging from 0.08 to 0.14 mM (ρ = 2.86 to 5.00), all curves displayed a similar featureless profile, denoting the absence of significant interactions between individual nanotubes (Figure 4A *left*). Still, the curves could be fitted by fractal aggregates with sizes over 2π/*q*_min_ ≈ 630 nm, with the minimum scattering vector *q*_min_ = 10^–3^ Å, probably due to the presence of some aggregates in solution scattering most of the signal. From [SPM^4+^] = 0.17 mM (ρ = 6.07) to 0.47 mM (ρ = 16.8), a drastic change was observed. Despite a signal of aggregation at low scattering vectors, all curves in Regime 2 could be fitted with aggregated polydisperse spheres (Figure 4A *middle*), denoting a 3D structuration of nanotubes in agreement with the formation of networks. Finally, for [SPM^4+^] ≥0.6 mM (ρ ≥ 21.3), a marked transition to Regime 3 was observed. This regime was characterized by a strong increase in scattering intensity and scattering curves that could be fitted by flat disks (Figure 4A *right*), denoting the formation of dense anisotropic objects, in agreement with the formation of bundles. Fits provided a disk thickness of around 30 nm, which was smaller yet comparable to the bundle diameter measured by TEM (Figure S1B). Notably, in this regime, we also detected a characteristic peak corresponding to a repeating distance of 2.5 to 3 nm, attributed to the interspacing DNA arrangement in the bundles, again in good agreement with the 3 nm value obtained from cryo-EM data (Figure 1C *right*). A similar succession of the three regimes was also observed using spermidine as the condensing agent (Figures S10 and S11), except that the transitions were observed at higher ρ for both Regime 1 – Regime 2 ([SPD^3+^] = 6.0 mM, ρ = 171) and Regime 2 – Regime 3 ([SPD^3+^] = 17 mM, ρ = 486) transitions. In Regime 3, fits of the SAXS data also provided a smaller disk thickness, in agreement with the decrease in bundle diameter observed and measured using TEM (Figure S6). The characteristic features of each regime allowed us to build a phase diagram exclusively established from the SAXS data (Figure 4B). We found that each of the nanotube assemblies (individual nanotubes, networks, and bundles) previously observed by fluorescence microscopy upon the addition of spermine or spermidine (Figure 3A) was obtained in the same range of charge ratio ρ with transition between regimes in remarkable agreement with the data obtained by SAXS (Figure S12). These results proved the consistency of the overall nanotube assembly behavior after the addition of multivalent condensing agents and demonstrated the transition from individual nanotubes with no organizational features to dense bundles with a high degree of internal organization composed of parallelly packed DNA strands with a characteristic repeating distance of 2.5 to 3 nm.

**Figure 4:**
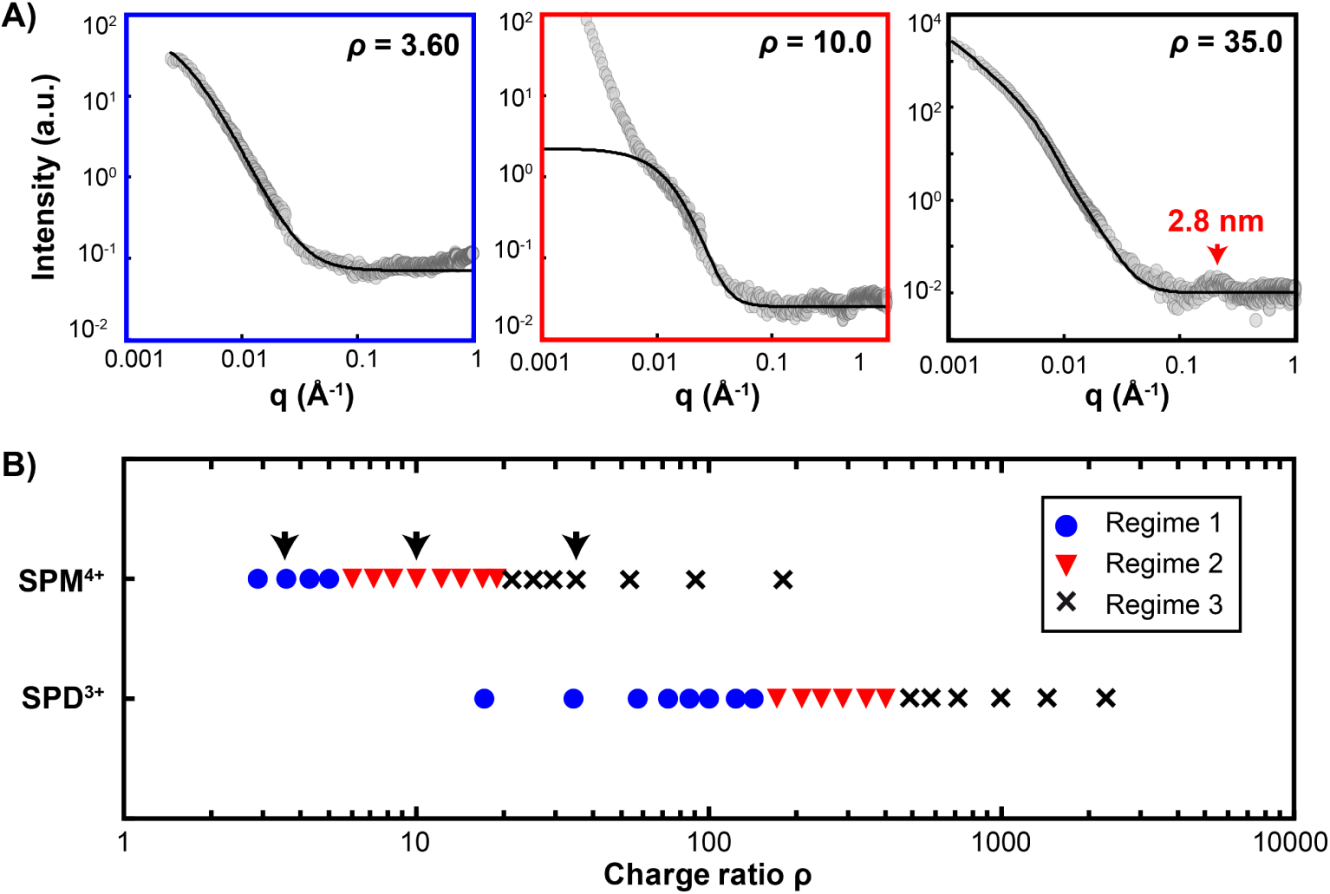
Small angle X-ray scattering reveals 3 regimes of interaction of nanotube assembly upon condensing agent addition. A) Representative scattering plots upon spermine addition for Regime 1 (ρ = 3.60), Regime 2 (ρ = 10.0), and Regime 3 (ρ = 35.0). Symbols are experimental points; solid black lines are fitting curves. At ρ = 35.0, the detected peak corresponds to a characteristic distance of 2.8 nm. B) Diagram showing the type of regime of nanotube assembly as a function of the charge ratio upon the addition of spermine (SPM^4+^) or spermidine (SPD^3+^). Black arrows correspond to the scattering plots shown in A. Each DNA strand concentration is 500 nM in TAMg buffer.

### Assembly in the Dilute Regime: Predominance of Ring Formation

The formation of DNA superstructures involving the interaction between many individual nanotubes, we then studied their assembly in a much more dilute regime upon spermine or spermidine addition (Figure 5). Using a 100-fold reduced DNA concentration (5 nM of each strand, Figure 5A *left*), we did not observe any interconnected structures, such as extended networks or highly clustered bundles (Figure 5A *right top*), but rather more individualized objects that could be adsorbed on glass slides for better scrutinization. Three characteristic structures could be rationally distinguished by image analysis of both the fluorescence intensity profile and morphology (see the Materials and Methods Section in Supporting Information for details): individual nanotubes (elongated shape, low fluorescence) and only two kinds of condensed structures, bundles (elongated shape, high fluorescence) and rings (toroidal shape, high fluorescence) (Figure 5A *right bottom*). Interestingly, due to the low DNA concentration, bundles were isolated, in contrast with the high concentration regime. We quantified the evolution of the structure distribution for increasing spermine concentration (Figure 5B). From [SPM^4+^] = 0.4 mM, individual nanotubes progressively disappeared to form the condensed structures, among which rings became predominant for [SPM^4+^] ≥ 0.8 mM. The ring diameter distribution was less broad and centered around slightly lower values than at high DNA concentration and was independent of both spermine concentration (Figure 5C) and condensing agent valency (Figure 5D). All these results show that in the dilute regime, adding multivalent condensing agents induces the formation of isolated condensed structures, with well-defined nanorings being preferentially formed to minimize surface and bending energy.

**Figure 5:**
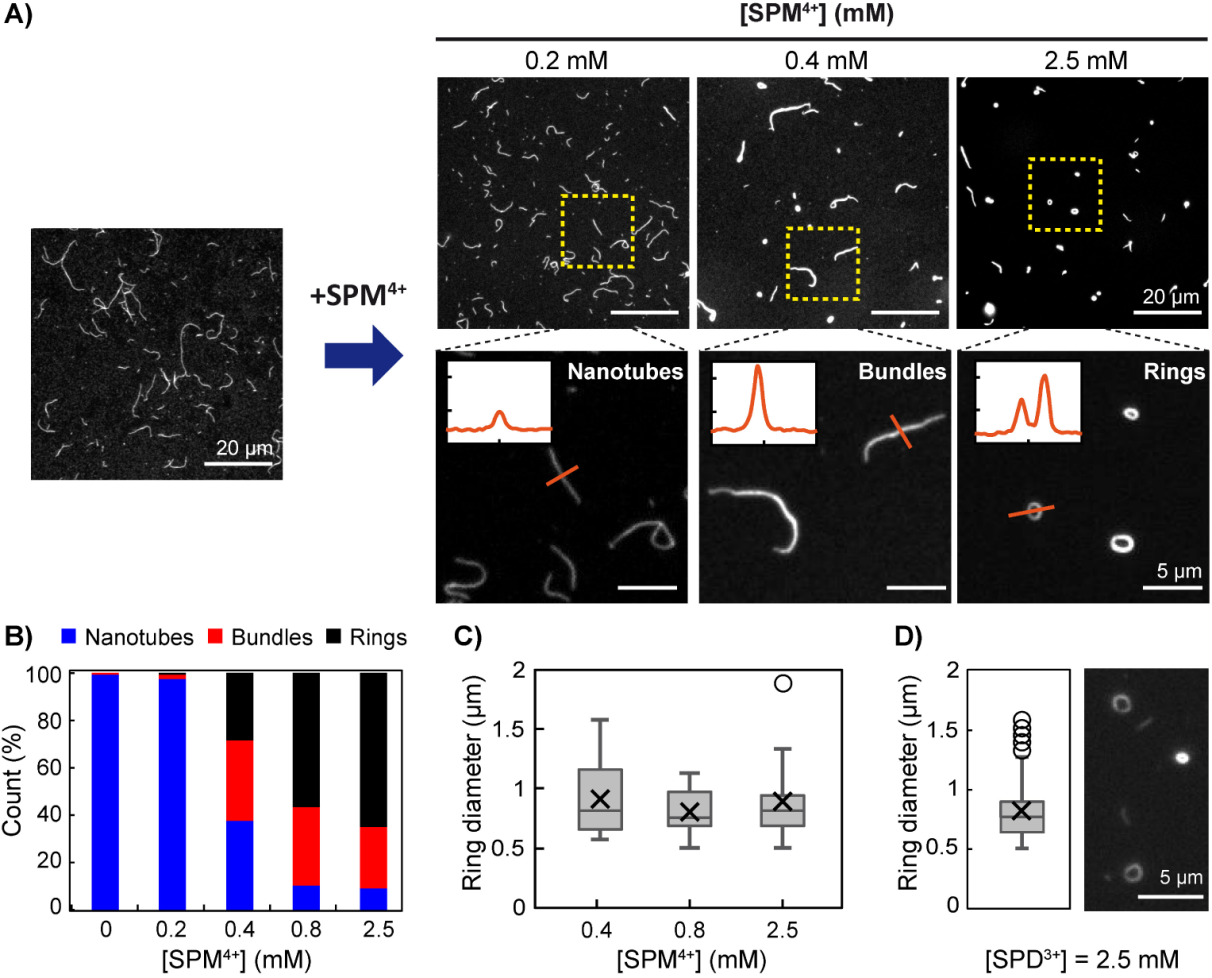
At low DNA concentration (5 nM per strand), spermine induces the formation of isolated bundles and well-defined rings, but no networks. A) Epifluorescence microscopy images of adsorbed structures as a function of spermine concentration (right top) added to nanotubes (left). Right bottom: enlarged images of characteristic structures observed above (nanotubes, bundles, or rings). Insets show the intensity profile (all displayed with the same scale, with *x* spanning 4 μm and *y* showing the fluorescence intensity (a.u.)) along the orange line crossing a given structure. B) Distribution (%) of adsorbed structures (individual nanotubes, bundles, or rings) as a function of spermine concentration (*n* = 440, 446, 107, 133, 113). C,D) Box and whiskers plots (outliers included) of the ring diameter distribution C) as a function of spermine concentration (*n* = 35, 47, 50), and D) after the addition of spermidine (2.5 mM, *n* = 86) with a representative epifluorescence microscopy image. The boxes range from the first to the third quartile, with the median and average indicated with a horizontal line and a cross, respectively. Each DNA strand concentration is 5 nM in TAMg buffer.

### Effect of DNA Concentration

Due to the differing behavior between the high DNA concentration and the dilute regimes, we systematically characterized the superstructure assembly upon spermine addition for various DNA concentrations ([DNA] is used to denote the concentration in each strand). For [DNA] = 100 and 50 nM, we observed a similar evolution as for 500 nM, with nanotubes first condensing into interconnected networks prior to forming clustered bundles (Figures S13 and S14). Notably, at [DNA] = 20 nM (Figure S15), we did not detect any interconnected structures (networks, clustered bundles) but observed instead an evolution similar to the situation at very low DNA concentration (5 nM, Figure 5). The transition between these two kinds of behavior is thus located at a concentration C* between 20 and 50 nM, in agreement with a dilute-to-semidilute regime transition. We estimated the theoretical value of C* as the strand concentration at which 1 nanotube occupies a sphere with a diameter of its size. Having measured an average size *L* of individual nanotube of around 7 μm, and considering nanotubes as straight cylinders with a number of tiles per nanotube cross-section *n*_tile_ and a longitudinal length for each tile of 14 nm,^24^ we obtained C* (strands·m^–3^) = 7000/14 × *n*_tile_/(4/3π × (3.5 × 10^–6^)^3^), that is, a value of C* ranging from 19 to 46 nM for previously reported values of *n*_tile_ (from 4 to 10), in good agreement with the experimentally observed transition. We then established the diagram of superstructure formation for various DNA concentrations as a function of the charge ratio (Figure 3B). The two characteristic evolutions were observed above and below C* (calculated here for an average *n*_tile_ value of 7). Notably, all transitions − nanotube-network, network-bundle, and nanotube-bundle− occurred at a charge ratio that increased with a decrease in DNA concentration. This could be explained by the fact that, due to the lower entropic gain upon spermine exchange with DNA counterions when decreasing the amount of DNA, a comparatively higher spermine concentration is necessary to reach the same neutralization and thus to induce nanotube assembly.

### Reversibility of Superstructure Formation

The reversibility of DNA condensation led us to explore whether the nanotube superstructures could be disassembled after their formation. It is known that adding an excess of monovalent cations on condensed DNA can compete with the DNA neutralization by multivalent counterions leading to DNA decompaction.^17,43^ Following this principle, nanotubes were first assembled into networks by introducing [SPM^4+^] = 0.4 mM prior to adding NaCl (100 mM) to the solution. After NaCl addition, nanotubes were observed to freely fluctuate in solution (Movie S2), and TEM revealed a majority of individual nanotubes showing no local organization, in a state comparable to that before spermine introduction (Figure 6A). To quantify the extent of nanotube condensation and recovery, we determined the surface density of adsorbed individual nanotubes. It was found to be significantly higher before spermine addition and after further Na^+^ addition (Figures 6B and S16), proving the efficiency of both the condensation of nanotubes upon spermine addition and their recovery by further Na^+^ addition. Compared to initial nanotubes, recovered ones appeared with a similar length distribution yet shifted to lower values with an average length of 7 ± 4 and 4 ± 3 μm for nanotubes before condensation and after recovery, respectively (Figure 6C). Similarly, the diameter distribution after recovery was similar to the initial state yet shifted to slightly higher values with an average diameter increasing from 12 ± 3 nm to 15 ± 2 nm (Figure 6D). These results show that nanotubes were efficiently recovered from their condensed state by adding an excess of monovalent cations and globally maintained their main structural features (length, diameter).

**Figure 6:**
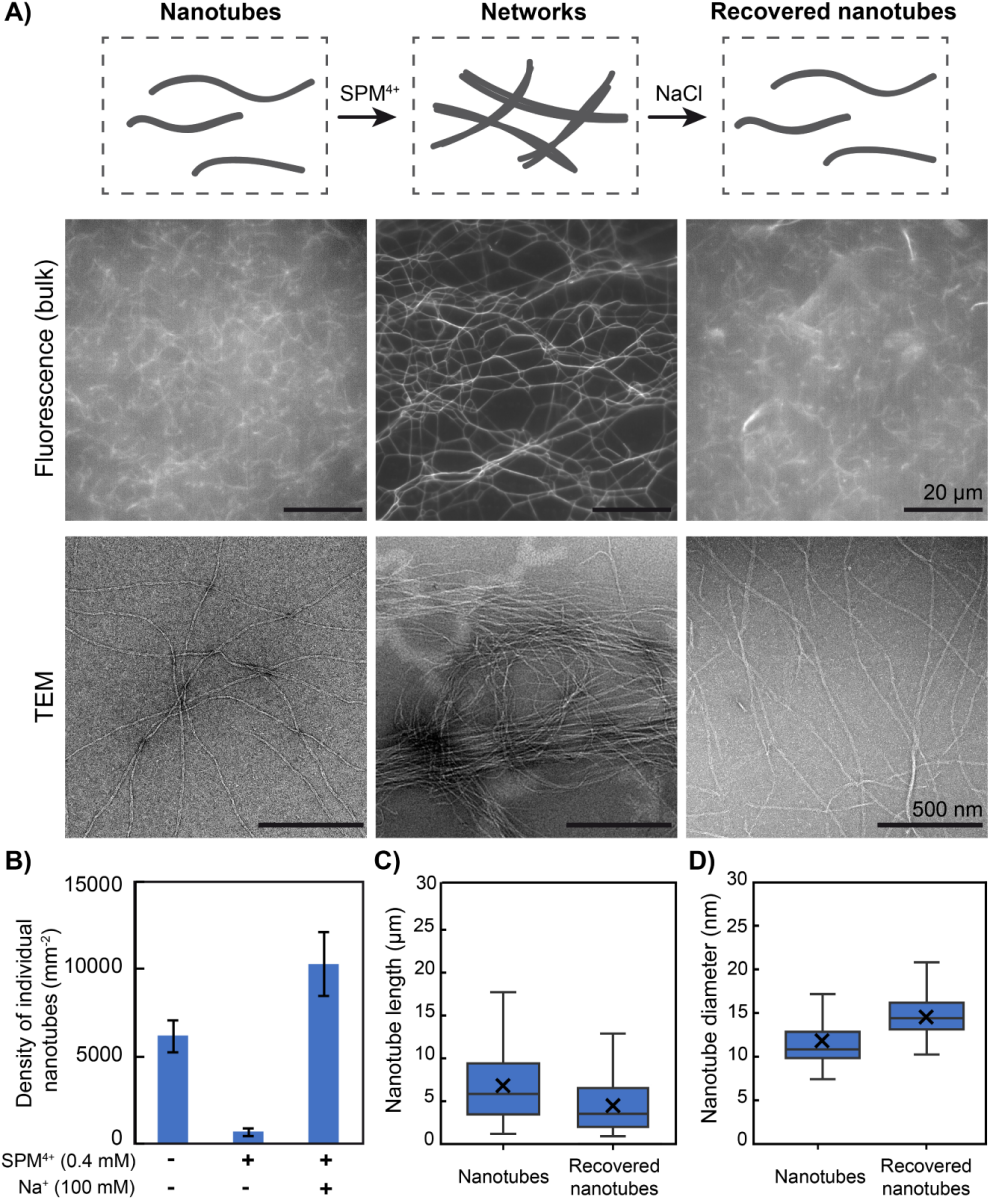
Reversible formation/disassembly of nanotube networks. Schemes (top), epifluorescence microscopy images (middle), and TEM images (bottom) of individual nanotubes (left), forming vast interconnected networks upon the addition of 0.4 mM spermine (middle), which can be disassembled upon further addition of 100 mM NaCl (right). B) Density of adsorbed individual nanotubes for the successive states shown in A. The error bars represent the standard deviation obtained on three images. C,D) Box and whisker plots (outliers excluded) of the C) length (*n* = 285, 226) and D) diameter (*n* = 45, 36) distributions of individual nanotubes before and after the successive addition of 0.4 mM spermine and 100 mM NaCl. Each DNA strand concentration is 500 nM in TAMg buffer.

To achieve further control with an external stimulus without having to change the chemical composition of the medium, we implemented AzoTAB, a photosensitive DNA-condensing agent.^44–47^ AzoTAB is a cationic amphiphilic molecule^48–51^ that neutralizes DNA in a photodependent manner,^47,52^ the *trans* isomer inducing DNA condensation at a lower concentration than the *cis* isomer. We used an AzoTAB solution kept in the dark (−UV, *trans*-rich state) or irradiated at 365 nm for 1 min (+UV, *cis*-rich state) (Figure 7A). Starting from individual nanotubes obtained in a TAMg buffer, we added increasing amounts of AzoTAB and studied the effect of UV irradiation in bulk solution (Figure 7B *top*). For [AzoTAB] = 0.1 mM, nanotubes were freely fluctuating in solution (Figure 7B *top left*) and appeared similar to nanotubes without AzoTAB (Figure 1B *left* and Movie S1). Increasing the AzoTAB concentration to 0.5 mM led to the formation of clustered nanotube assemblies (Figure 7B *top middle*) similar to the networks obtained with multivalent cations. Notably, for the same AzoTAB concentration with UV irradiation, nanotubes did not form any interconnected networks but remained fluctuating in solution (Figure 7B *top right*). To further characterize this photodependent assembly behavior, we analyzed the structures obtained by adsorption on a glass surface after dilution (Figure 7B *bottom*). With increasing concentration of AzoTAB from 0.1 to 0.5 mM without UV, the adsorbed structures appeared in lower numbers and with a high fluorescence intensity profile, which was a sign of associative interactions between nanotubes. At the same concentration with UV, the structures appeared with a low intensity profile in a state similar to that of low concentration [AzoTAB] = 0.1 mM. We determined the density of adsorbed individual nanotubes identified as elongated structures displaying a low intensity profile and showed that the density significantly increased when UV irradiation was applied (Figure 7C). This showed that for [AzoTAB] = 0.5 mM, we can control the assembly of nanotubes into either free individual nanotubes (+UV) or superstructures (−UV) in a photodependent manner (Movie S3). This creates ground for dynamic photoactuation of nanotube higher-order organization and superstructure formation.

**Figure 7:**
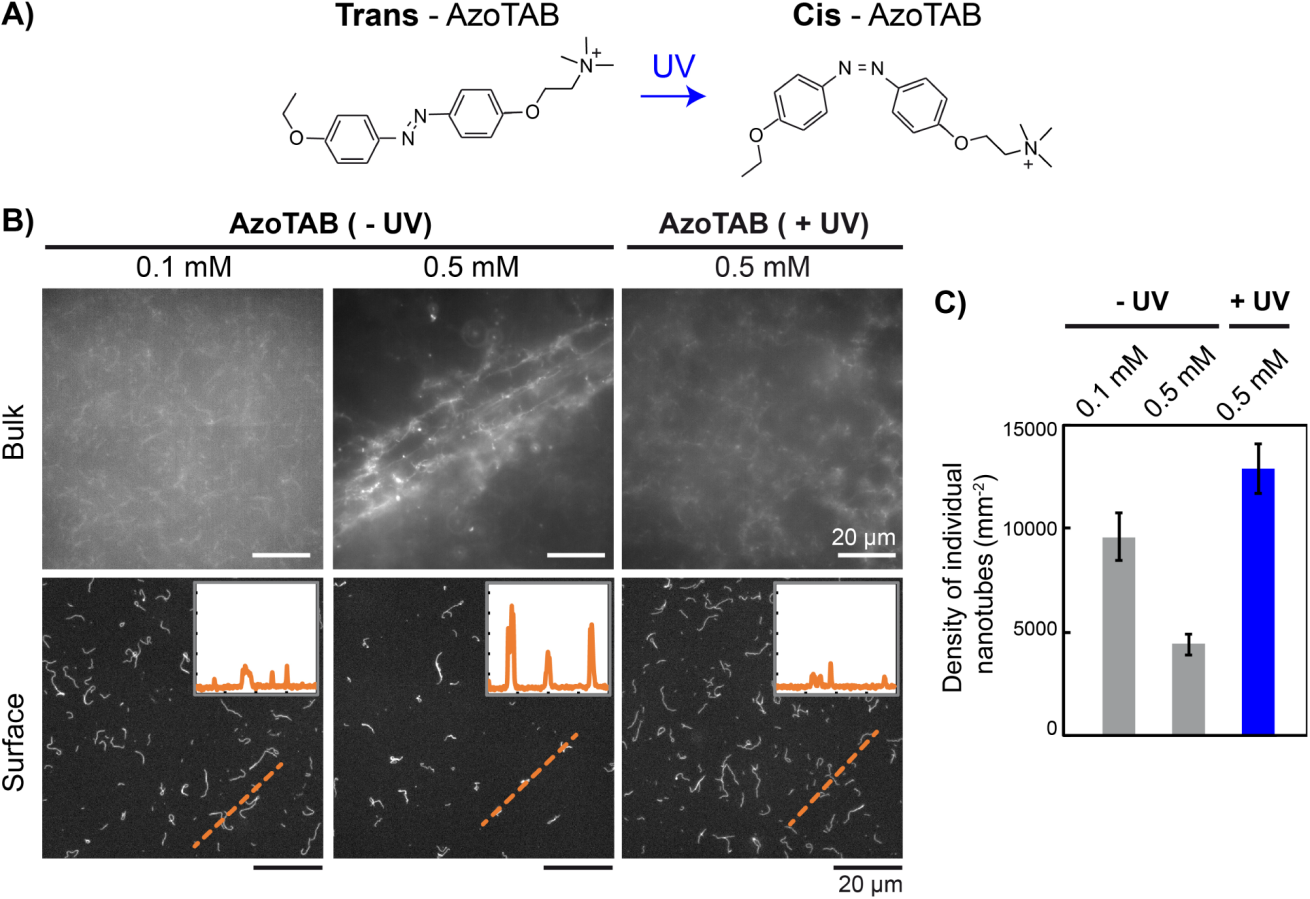
Photocontrol of superstructure formation using the photosensitive DNA condensing agent AzoTAB. A) *Trans*- to *cis*-photoisomerization of AzoTAB upon UV irradiation at 365 nm. B) Epifluorescence microscopy images of individual nanotubes or DNA superstructures obtained for different AzoTAB concentrations, without (−UV) or after (+UV) irradiation at 365 nm for 1 min, in the bulk (top) or adsorbed after a 100-fold dilution (bottom). Insets show the intensity profile (all displayed with the same scale, with *x* spanning 40 μm and *y* showing the fluorescence intensity (a.u.)) along the orange dashed line crossing adsorbed structures. C) Density of adsorbed individual nanotubes for the conditions in part B. The error bars represent the standard deviation obtained on three images.

## Conclusion

We have shown that self-assembled DNA nanotubes of micrometric length condense into various higher-order structures upon the addition of sufficient amounts of multivalent cations, such as Mg^2+^, the triamine spermidine, and the tetraamine spermine. Based on electrostatic interactions, the approach offers a way to organize preassembled DNA nanostructures (here, nanotubes) into superstructures in a base-pairing-orthogonal and sequence-independent way. Compared to Watson–Crick–Franklin-based assembly methods, such as DNA origami or single-stranded tiles (SSTs), it offers less programmability as well as a smaller repertoire of attainable morphologies. However, it has the advantage of being able to produce structures on a wide range of dimensions, from nano- and microscale (rings, bundles) to macroscale (extended networks), whereas programmable DNA assemblies are generally limited to a specific size range (for instance, around 100 nm in the case of DNA origami) unless specific protocols are applied. Moreover, the method is very fast, with superstructures being obtained in a subminute timescale, to be compared with the hours to days typically required for DNA origami, nanotubes, or SST assemblies. It is also universal, the same condensing agent being applicable to virtually any DNA structure or sequence. Finally, it offers the possibility for disassembly at constant temperature by the simple addition of monovalent cations. We demonstrated the strong analogy with the phenomenology of DNA condensation, through (i) the crucial role of counterion valency (the higher the valency, the lower the charge ratio required to induce a transition) and (ii) the formation of rings made of parallelly packed DNA strands and resembling the toroids formed by the condensation of giant double-stranded DNA. We also evidenced an important role of DNA concentration, with two different behaviors below and above a characteristic concentration C* representing the overlapping of nanotubes. Below C*, nanotubes are in a dilute regime, and adding DNA condensing agents led to the formation of well-defined nanorings or isolated bundles. Above C*, interconnected structures were formed such as clustered bundles and, notably, vast 3D networks at intermediate DNA condensing agent concentrations. In terms of the structures obtained, the system also recapitulated some of the known features obtained with other semiflexible or rigid polyelectrolytes such as actin filaments and microtubules, with the formation of networks and bundles, thus reinforcing knowledge of the electrostatic assembly of polyelectrolytes as well as opening perspectives for building cytoskeleton-inspired materials. Mainly driven by electrostatics, the assembly principles explored here provide a ubiquitous basis for the formation of superstructures independent of their detailed chemistry. It is therefore not only a means of building superstructures that combine nanoscale DNA programmability and higher-order organization, but also a strategy for organizing bricks other than DNA. The superstructure disassembly by monovalent salt addition and the photocontrol enabled in the presence of photosensitive DNA condensing agents highlight some useful principles for reconfigurable DNA assembly and constitute ground for the elaboration of highly dynamic, multiscale DNA-based smart materials.

## Supplementary Material









## Notes

Materials and methods; Supplementary Text S1 (charge ratio); Supplementary Figures S1 (Distributions of individual nanotubes and nanotube bundles diameters), S2 (Phase diagram as a function of DNA concentration), S3–S5 (Epifluorescence images of DNA structures and superstructures as a function of condensing agents concentration), S6 (TEM analysis of nanotube bundles formed by spermidine addition), S7 (Epifluorescence images of DNA structures and superstructures formed in TANa buffer), S8 (Distribution of ring diameters in TANa buffer), S9–S10 (X-ray scattering curves upon addition of spermine and spermidine), S11 (Representative scattering plots upon spermidine addition), S12 (Comparison of phase diagrams obtained by fluorescence microscopy and SAXS), S13–S15 (Epifluorescence images of DNA structures and superstructures for different DNA concentrations), S16 (Epifluorescence images of adsorbed DNA structures during the assembly/disassembly process) (PDF)
Movie S1 (Epifluorescence microscopy observation of the formation of DNA networks or bundles) (MP4)
Movie S2 (Epifluorescence microscopy observation of the reversible formation and dissociation of DNA networks using spermine and NaCl) (MP4)
Movie S3 (Epifluorescence microscopy observation of the photosensitive formation of DNA networks induced by the addition of AzoTAB) (MP4)
