# Supplementary material for: DNA Condensation-Inspired Assembly of DNA Nanotubes into Reversible Superstructures: A Base Pairing-Orthogonal Way to Create Rings, Bundles, or Vast Networks
Source: J Am Chem Soc. 2025 Oct 1;147(41):37317–27. doi: 10.1021/jacs.5c10921 (PMC12532283; doi:10.1021/jacs.5c10921)
Supplement: Supplementary file 1 [file ja5c10921_si_001.pdf]

Supplementary Information for:

DNA condensation-inspired assembly of DNA nanotubes into reversible superstructures: a base pairing-orthogonal way to create rings, bundles or vast networks

Laura Bourdon,[1] Xiang Zhen Xu,[2] Laurent J. Michot,[3] Mathieu Morel,[1] Sergii Rudiuk,[1] Ayako Yamada,[1] Damien Baigl[1]*

[1]CPCV, Department of Chemistry, École Normale Supérieure, PSL University, Sorbonne Université, CNRS, 75005 Paris, France

[2]Laboratoire de Physique et d'Etude des Matériaux (LPEM), CNRS UMR 8213, ESPCI-Paris, PSL Research University, Sorbonne Université, 10 rue Vauquelin, Paris, 75005 France

[3]Laboratory of Physical Chemistry of Electrolytes and Interfacial Nanosystems (PHENIX), UMR 8234 CNRS, Sorbonne University, Paris 75005, France

*correspondence to: damien.baigl@ens.psl.eu

Contents

Materials and methods

Supplementary Text S1

Supplementary Figures S1–S16

Legends of the Supplementary Movies S1–S3

Supplementary references

2. Materials and methods

Materials

All the DNA oligonucleotides, whose sequences and modifications are presented below, were purchased from Sigma with a PAGE purification. They were resuspended in MQ water at a concentration of 20 µM, aliquoted and stored at -20°C until further use. The salts (magnesium chloride and sodium chloride), the polyamines (spermidine and spermine) and the AzoTAB were purchased from Sigma and were dissolved in MQ water as working solutions.

Oligonucleotide sequences

The sequences of the five DNA strands composing the nanotubes were taken from the article [1] and are listed below with the same nomenclature (5' to 3'). The third strand has a Cy3 dye on its 5' extremity.

5bSE1	CTCAGTGGACAGCCGTTCTGGAGCGTTGGACGAAACT
5bSE2	GTCTGGTAGAGCACCACTGAGAGGTA
5bSE3-Cy3	Cy3 – CCAGAACGGCTGTGGCTAAACAGTAACCGAAGCACCAACGCT
5bSE4	CAGACAGTTTCGTGGTCATCGTACCT
5bSE5	CGATGACCTGCTTCGGTTACTGTTTAGCCTGCTCTAC

Self-assembly of DNA nanotubes

The DNA nanotubes were assembled according to the original protocol [1]. The five DNA strands were mixed in a buffered solution (40 mM Tris-Acetic acid pH 8) containing either 12.5 mM $MgCl_2$ (TAMg) or 100 mM NaCl (TANa) to a final concentration of 500 nM each. The solution was heated at 90°C and cooled down to 25°C with a ramp of -1°C/5 min. The annealed solution was used and characterized the next day.

Effect of DNA concentration

For studies performed at a DNA concentration lower than 500 nM in each strand, a DNA nanotube solution was prepared at 500 nM following the above protocol prior to dilution at the desired concentration using the same buffer.

Formation of DNA superstructures

The nanotubes and the formation of DNA superstructures were observed in custom made wells of 3 mm diameter. These wells were obtained by placing a punched PDMS layer onto a glass coverslip. To form the DNA superstructures in the wells, we added 20 µL of nanotube solution and a small volume (< 5% of the total volume) of $MgCl_2$, NaCl, spermidine or spermine concentrated solution. After each addition, the solution was gently mixed by two aspirations/refluxes of a 20 µL pipette, then observed under the microscope.

Epifluorescence microscopy

The DNA nanotubes and the DNA superstructures were observed inside the wells, or on the surface of a glass cover slip, using a Zeiss Observer Z1 microscope equipped with a Plan-Apochromat 100x/1.4 NA oil objective, and an Andor Zyla camera.

Confocal and super-resolution microscopy

An Andor BC43 Benchtop Confocal Microscope (Oxford Instruments) equipped with a sCMOS camera, was used to observe the DNA bundles obtained from DNA nanotubes and the addition of [SPM^{4+}] = 2.5 mM. A software-based Andor super-resolution module (Oxford Instruments) allowed us to get an image of a DNA ring (**Fig. 2C**) with improved lateral resolution down to ~140 nm.

Transmission Electron Microscopy (TEM)

The individual nanotubes or superstructures were deposited on a plasma-treated 200 mesh copper grid which supports a carbon film (Ted Pella, USA): 10 µL of solution was added on the grid and left for 3 minutes before blotting it with a filter paper. The sample was negatively-stained using Uranyl acetate (UA): 5 µL of 2% UA solution was added and quickly blotted, then 15 µL UA solution was again added and left for 1 minute before a last blotting. The samples were observed at 200 kV using a JEM-2010F microscope (JEOL) equipped with a field emission gun (FEG), a ultra-high resolution objective and a Rio 1816B camera (GATAN).

Cryo-electron microscopy (Cryo-EM)

We analyzed one 20-fold diluted solution of DNA nanotubes (each DNA strand was 25 nM), and one solution of DNA bundles that was obtained from 5-fold diluted nanotube solution (each strand was 100 nM) and the addition of 2.5 mM spermine. 4 µL of DNA solutions were

deposited on glow-discharged carbon-formvar lacey grids (Ted Pella, USA), blotted from the back side and flash frozen in liquid ethane with an EM-GP2 Leica plunger at 80% humidity. Cryo-EM images were acquired with a Glacios cryo-electron microscope (Thermofisher, USA) operating at 200 kV with a falcon IV camera and in low dose mode.

Small-Angle X-ray Scattering (SAXS)

SAXS experiments were carried out on beamline SWING at synchrotron SOLEIL (Saint-Aubin, France). The incident energy was fixed at 12keV and two sample-to-detector distances of 0.5 m and 6.2 m were used, yielding a q range (where $q = 4\pi\sin\theta/\lambda$ with θ, half the diffusion angle and λ the wavelength) extending from 10^{-3} Å^{-1} to 2 Å^{-1}. Nanotube solutions (each DNA strand was 500 nM) containing either SPM^{4+} or SPD^{3+} were conditioned in 1 mm diameter glass capillaries. The scattering curves obtained were corrected by subtracting the signal of a capillary containing TAMg buffer.

Reversible assembly/disassembly of DNA networks

20 µL DNA nanotubes (each strand was 500 nM) were introduced in a well and condensed into networks by adding 0.83 µL of 10 mM spermine solution (final concentration of spermine is 0.4 mM) and mixing by pipet aspirations/refluxes. After observing the networks using epifluoresence microscopy, 0.45 µL of 5 M NaCl solution was added to the same solution and mixing again by pipet aspirations/refluxes. The solution was observed one more time using epifluorescence microscopy. To quantify the density of individual nanotubes at each of these three steps (**Fig. 6B**), we post-diluted the solutions 100-fold using the corresponding buffer (TAMg, TAMg containing 0.4 mM SPM^{4+}, or TAMg containing 0.4 mM SPM^{4+} and 100 mM NaCl) and adsorbed them onto a glass surface before imaging by epifluorescence microscopy.

Photostimulation

40 µL stock solution of 10 mM AzoTAB was split into two 0.5 mL tubes. One of these solutions was placed under 365 nm irradiation for 1 min with an intensity of 334 mW·cm^{-2} (Precis Excite from CoolLED) measured with a powermeter (PM100D, Thorlabs GmbH). In a well containing 20 µL of DNA nanotubes (each strand was 500 nM), we added a small volume (< 5% of the total volume) of either the AzoTAB solution that was not exposed to the 365 nm light (- UV) or the one that was exposed (+UV). After mixing by pipetting, we observed the solution using

epifluorescence microscopy. To quantify the density of individual nanotubes (**Fig. 7C**), we diluted each nanotube solution 100-fold using the corresponding buffer (TAMg containing AzoTAB exposed or not under UV light) and adsorbed them onto a glass surface before imaging by epifluorescence microscopy.

Measurements of lengths and diameters

Measurements of structure sizes were performed on epifluorescence microscopy (nanotube length and ring diameter) and TEM (nanotube and bundle diameter) images using ImageJ software. The perimeter-equivalent diameter of rings, usually referred to as "diameter" in the main text, was obtained by measuring the perimeter of the closest ellipse divided by π. Nanotube lengths were measured by drawing a freehand line, and nanotube and bundle diameters were measured by drawing a wide straight line connecting the two edges of the elongated structures.

Distribution and density of adsorbed DNA structures

Each distribution (**Fig. 5B**) and density (**Figs. 6B and 7C**) of adsorbed individual nanotubes, bundles and rings was performed on three epifluorescence microscopy 128.87x128.87 μm images, counting each structure using the Image J software's multi-point selection tool. Bundles and rings were distinguished from individual nanotubes by their high fluorescence intensity profile.

2. Supplementary Text

Text S1. Determination of the charge ratio.

The charge ratio was ratio ρ was calculated as follows:

$$\rho = \frac{C_{cation}\zeta_{cation}}{C_{tile}n_{tile}}$$

where C_{cation} is the concentration of cations added to DNA nanotubes, $\zeta_{cation}$is the cation charge, C_{tile} is the concentration of tile in solution (500 nM), and n_{tile} is the number of nucleic bases contained in one DNA tile (n_{tile} = 168).

ζ_{cation} was calculated as follows:

$$\zeta_{magnesium} = 2$$

$$\zeta_{spermidine} = \frac{3 + 2 \times 10^{pH-pK_1} + 10^{2pH-pK_1-pK_2}}{1 + 10^{pH-pK_1} + 10^{2pH-p\ {}_1-pK_2} + 10^{3pH-p\ {}_1-pK_2-pK_3}}$$

With pK_1 = 8.24; pK_2 = 9.81; pK_3 = 10.89 for spermidine.[1,2]

$$\zeta_{spermine} = \frac{4 + 3 \times 10^{pH-pK_1} + 2 \times 10^{2pH-pK_1-pK_2} + 10^{3pH-pK_1-pK_2-pK_3}}{1 + 10^{pH-pK_1} + 10^{2pH-pK_1-pK_2} + 10^{3pH-pK_1-pK_2-pK_3} + 10^{4pH-pK_1-pK_2-pK_3-pK_4}}$$

With pK_1 = 7.96; pK_2 = 8.85; pK_3 = 10.02; pK_4 = 10.8 for spermine.[1,2]

3. Supplementary Figures

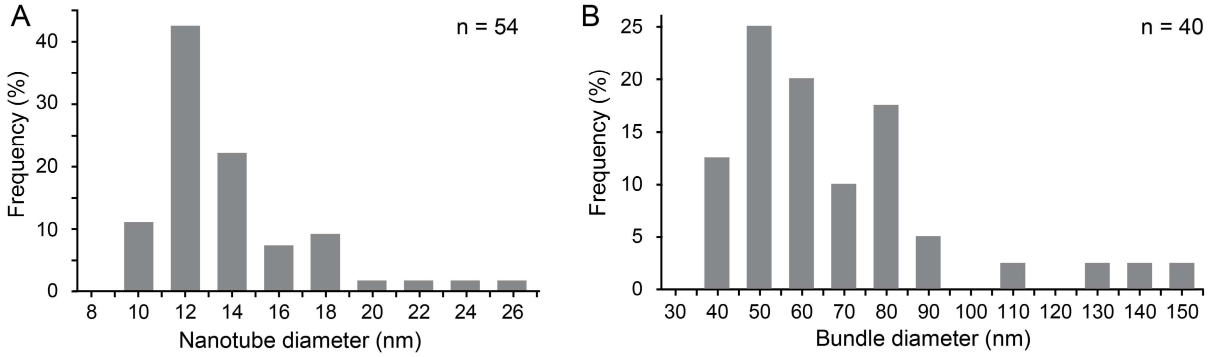

Figure S1. Distributions of diameter for A) individual nanotubes and for B) nanotube bundles formed with the addition of [SPM^{4+}] = 2.5 mM. Each DNA strand concentration is 500 nM in TAMg buffer.

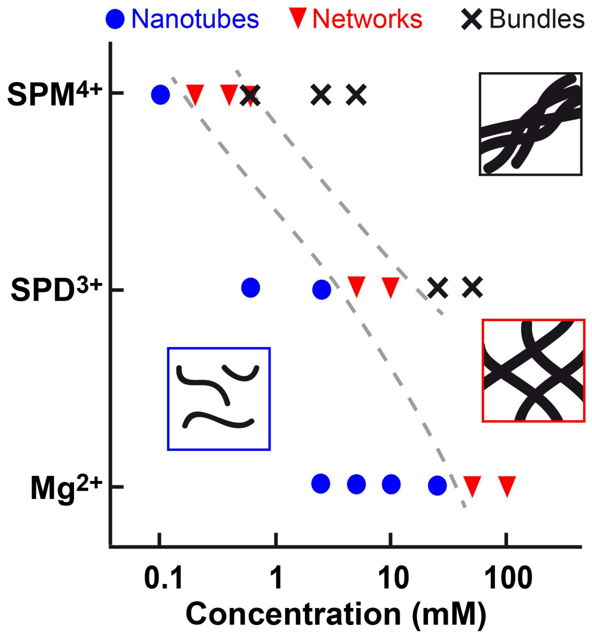

Figure S2. Diagram showing the superstructures formed by the nanotubes after addition of different multivalent cations (magnesium Mg^{2+}, spermidine SPD^{3+} or spermine SPM^{4+}), as a function of their concentration. Each DNA strand concentration is 500 nM in TAMg buffer.

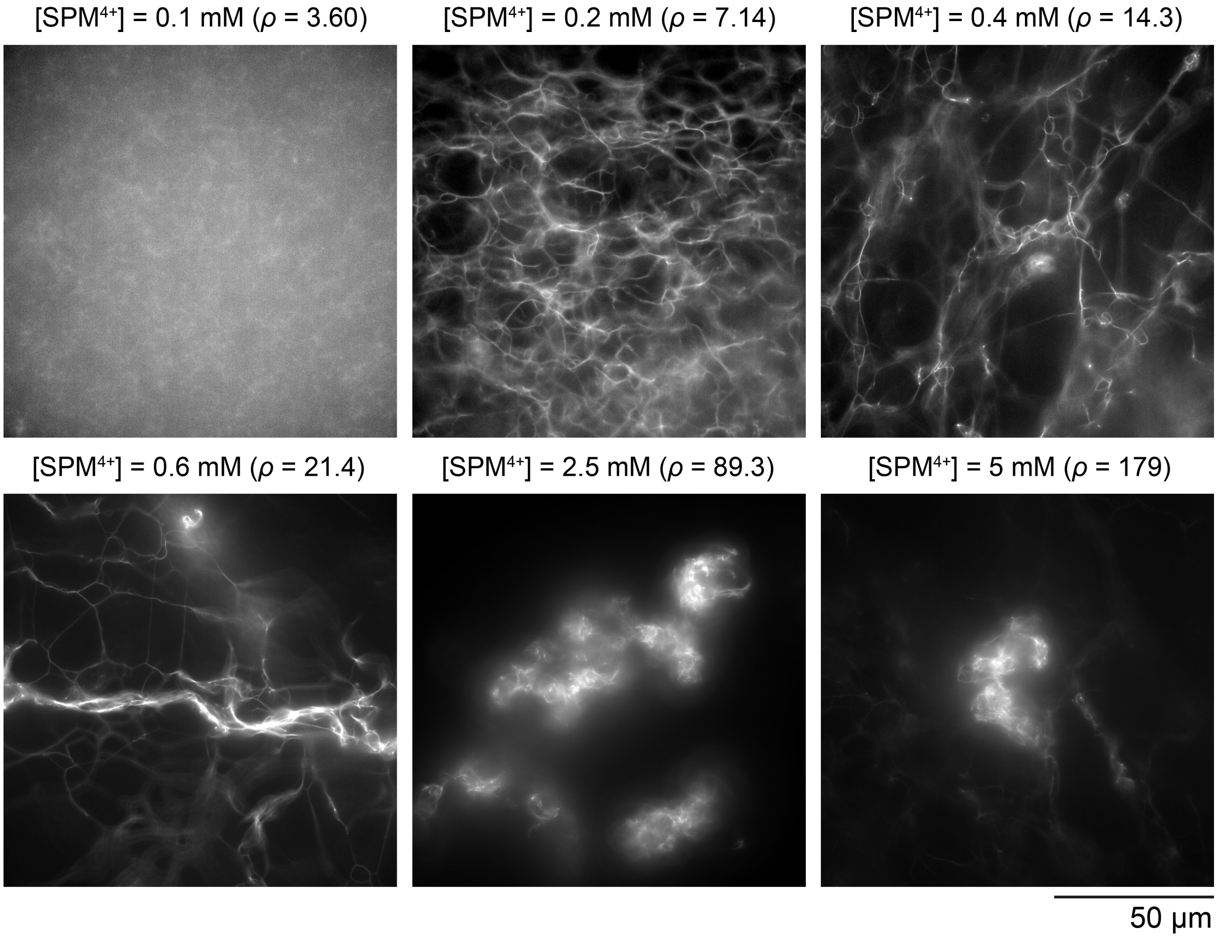

Figure S3. Epifluorescence images of DNA structures and superstructures obtained after the addition of different amount of spermine (SPM^{4+}) to individual DNA nanotubes. Each DNA strand concentration is 500 nM in TAMg buffer.

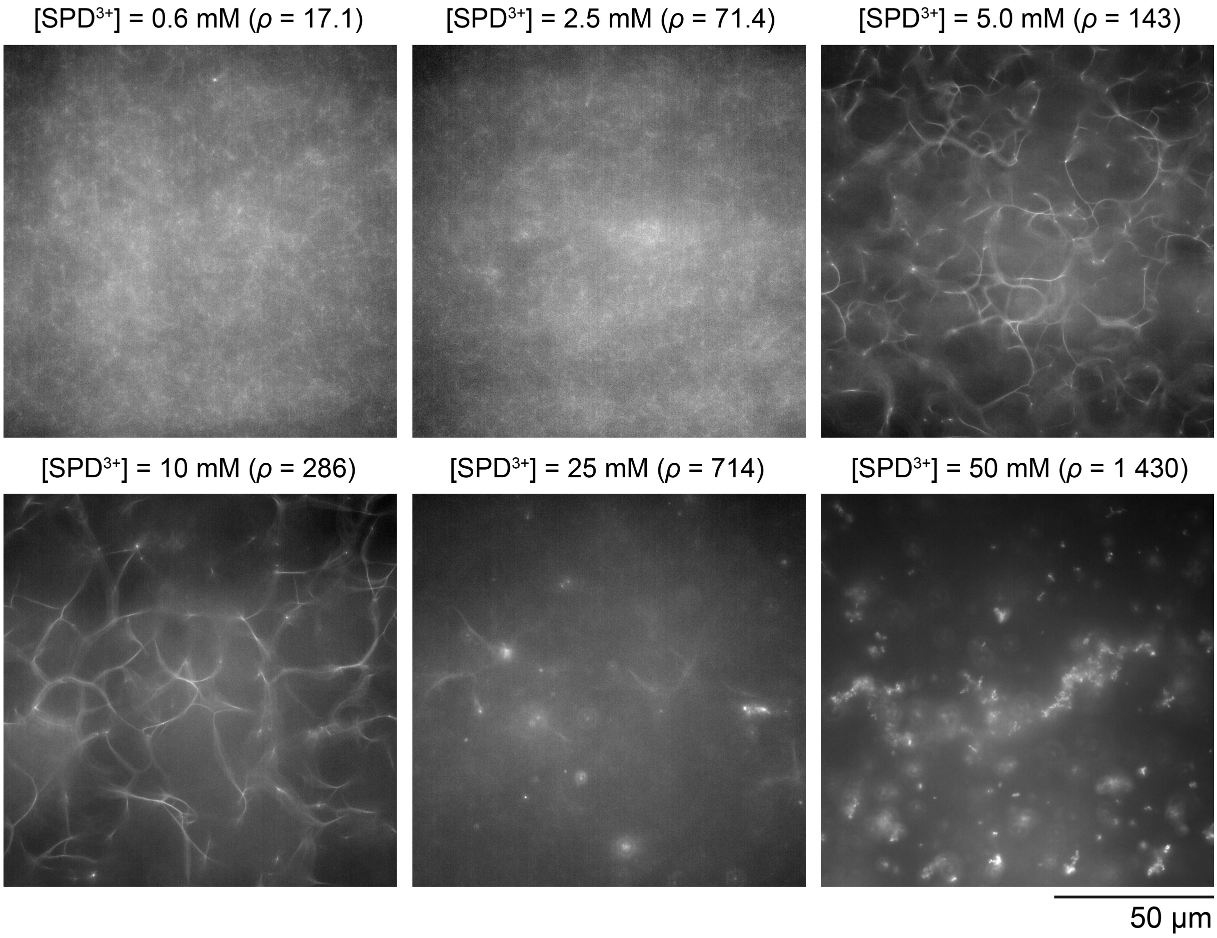

Figure S4. Epifluorescence images of DNA structures and superstructures obtained after the addition of different amount of spermidine (SPD^{3+}) to individual DNA nanotubes. Each DNA strand concentration is 500 nM in TAMg buffer.

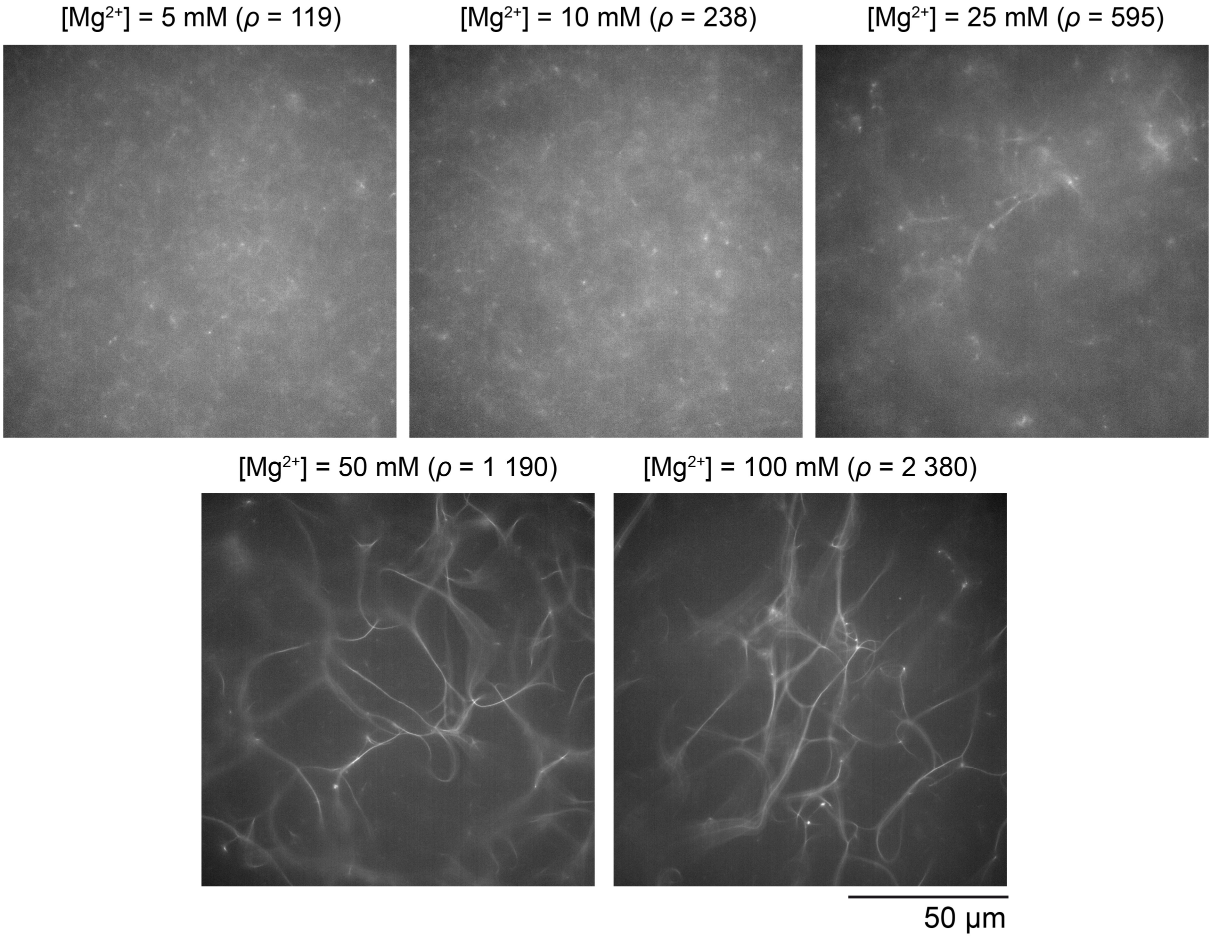

Figure S5. Epifluorescence images of DNA structures and superstructures obtained after the addition of different amount of $MgCl_2$ to individual DNA nanotubes. Each DNA strand concentration is 500 nM in TAMg buffer.

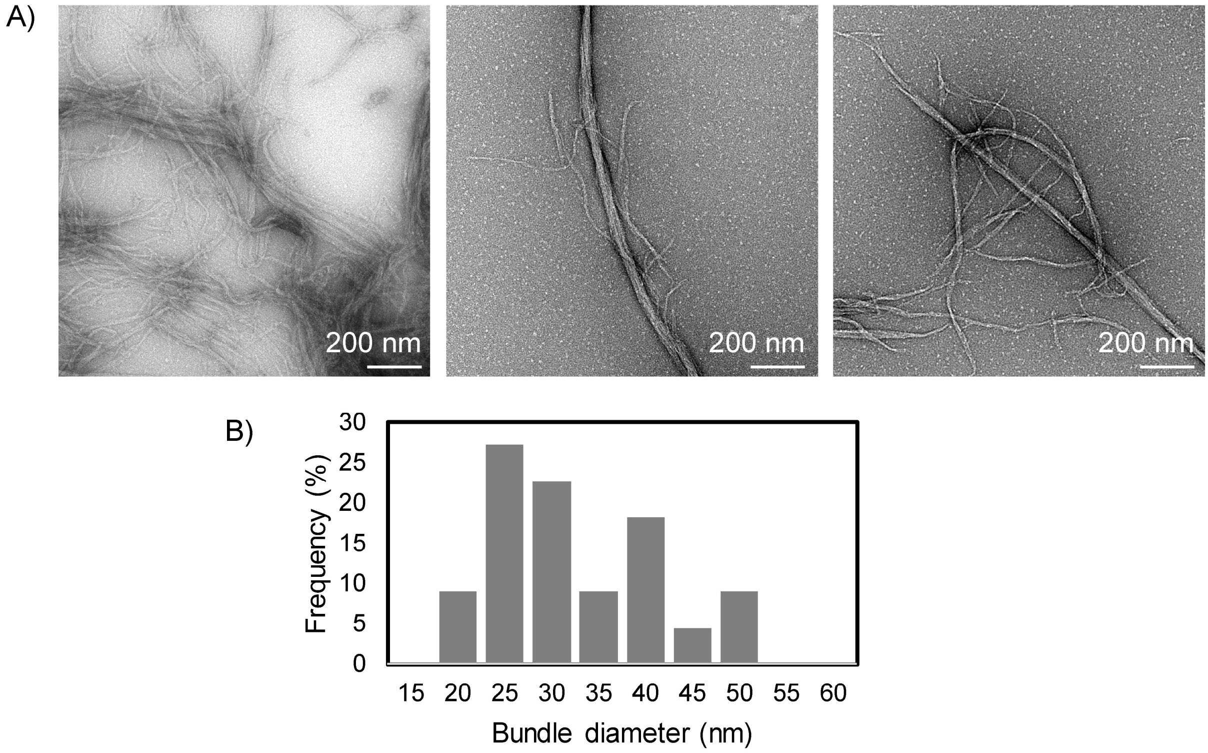

Figure S6. A) Transmission electron microscopy (TEM) images and B) diameter distribution of nanotube bundles formed with the addition of spermidine ([SPD^{3+}] = 50 mM, ρ = 1430). Each DNA strand concentration is 500 nM in TAMg buffer.

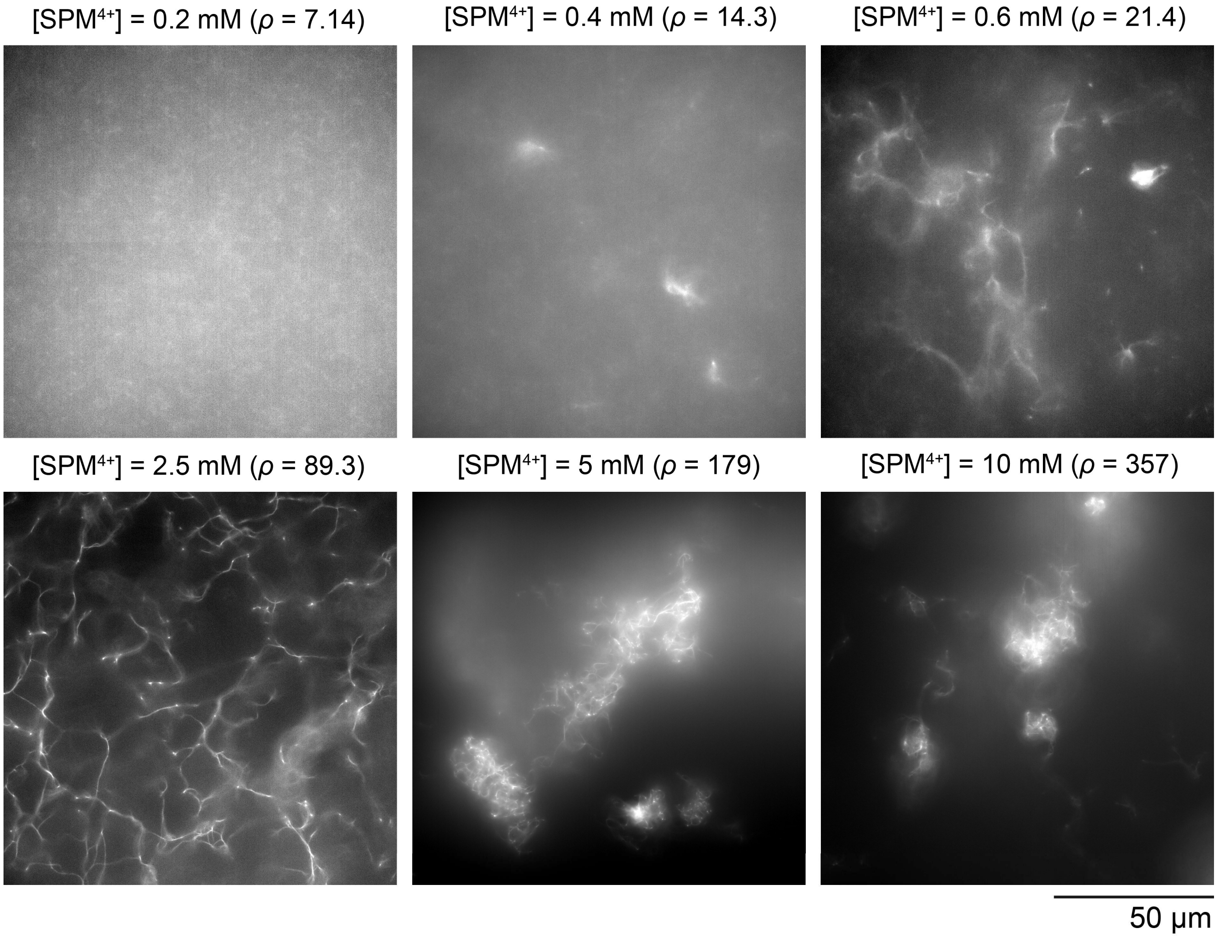

Figure S7. Epifluorescence images of DNA structures and superstructures obtained after the addition of different amount of spermine (SPM^{4+}) to individual DNA nanotubes assembled in TANa ([NaCl] = 100 mM). Each DNA strand concentration is 500 nM.

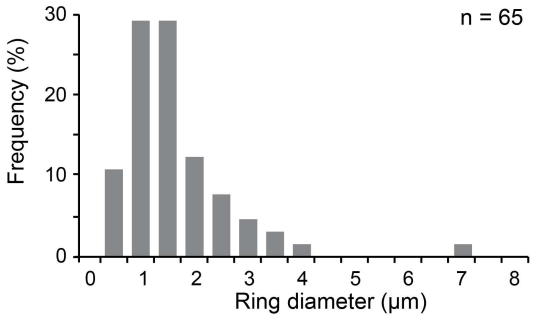

Figure S8. Distribution of the ring diameters obtained by condensing nanotubes formed in TANa ([NaCl] = 100 mM) using 12.5 mM SPM^{4+}. Each DNA strand concentration is 500 nM.

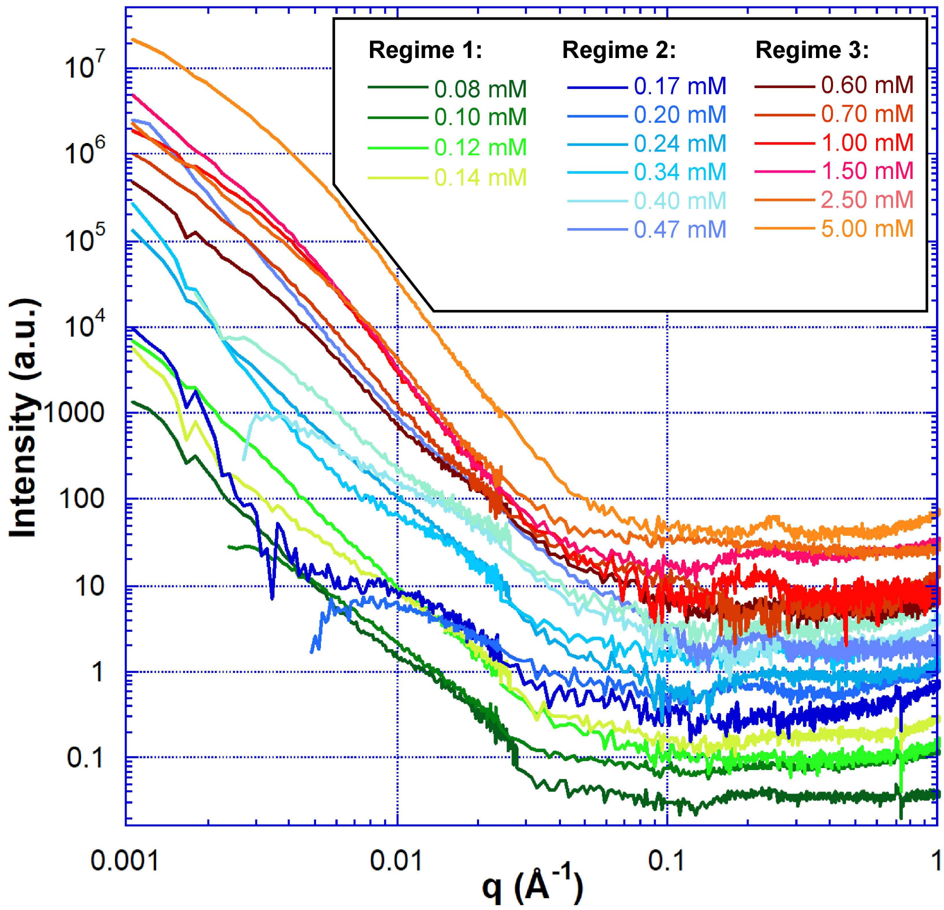

Figure S9. X-ray scattering curves of DNA nanotubes after the addition of different amount of SPM^{4+}. Each DNA strand concentration is 500 nM in TAMg buffer.

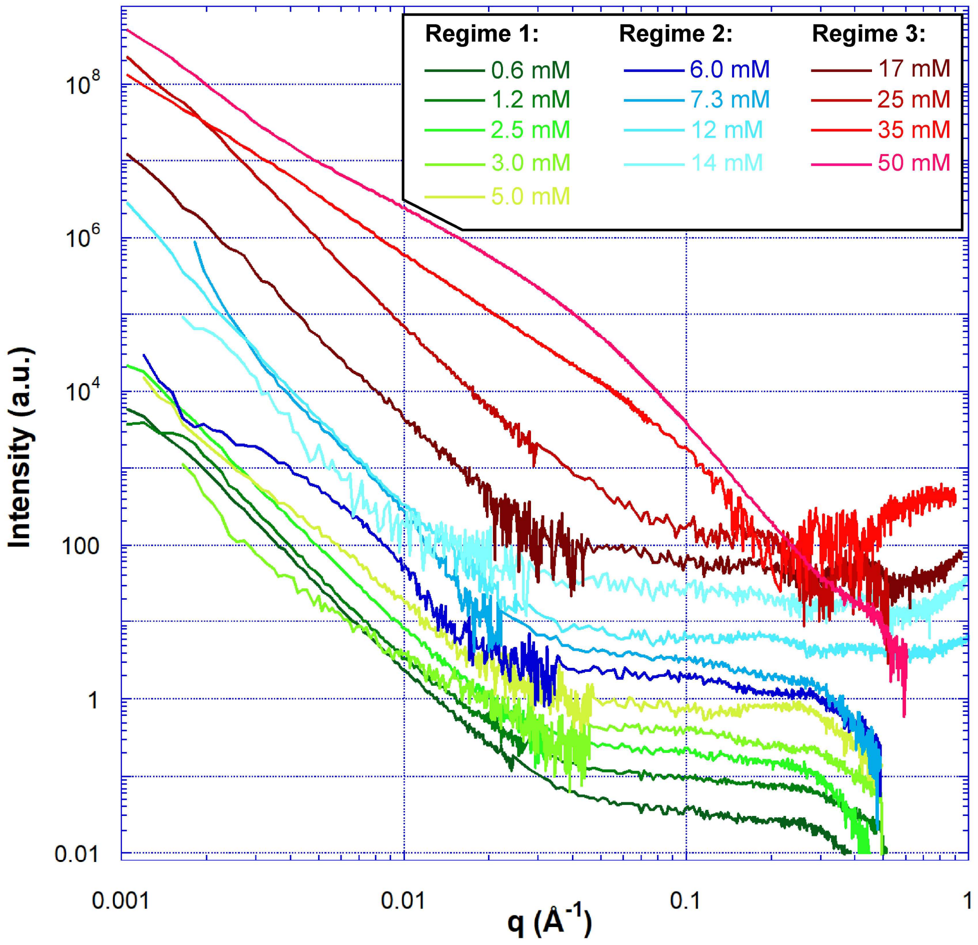

Figure S10. X-ray scattering curves of DNA nanotubes after the addition of different amount of SPD^{3+}. Each DNA strand concentration is 500 nM in TAMg buffer.

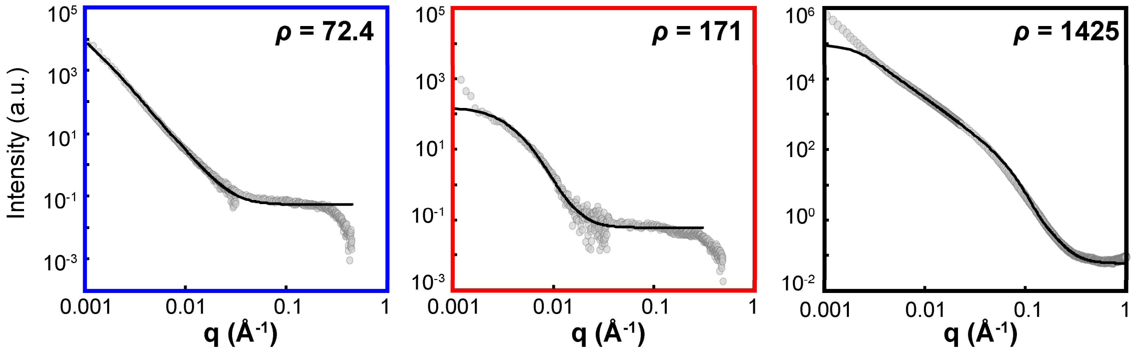

Figure S11. Representative scattering plots upon spermidine addition for Regime 1 (ρ = 72.4), Regime 2 (ρ = 171) and Regime 3 (ρ = 1425). Symbols are experimental points; solid green lines are fitting curves. Each DNA strand concentration is 500 nM in TAMg buffer.

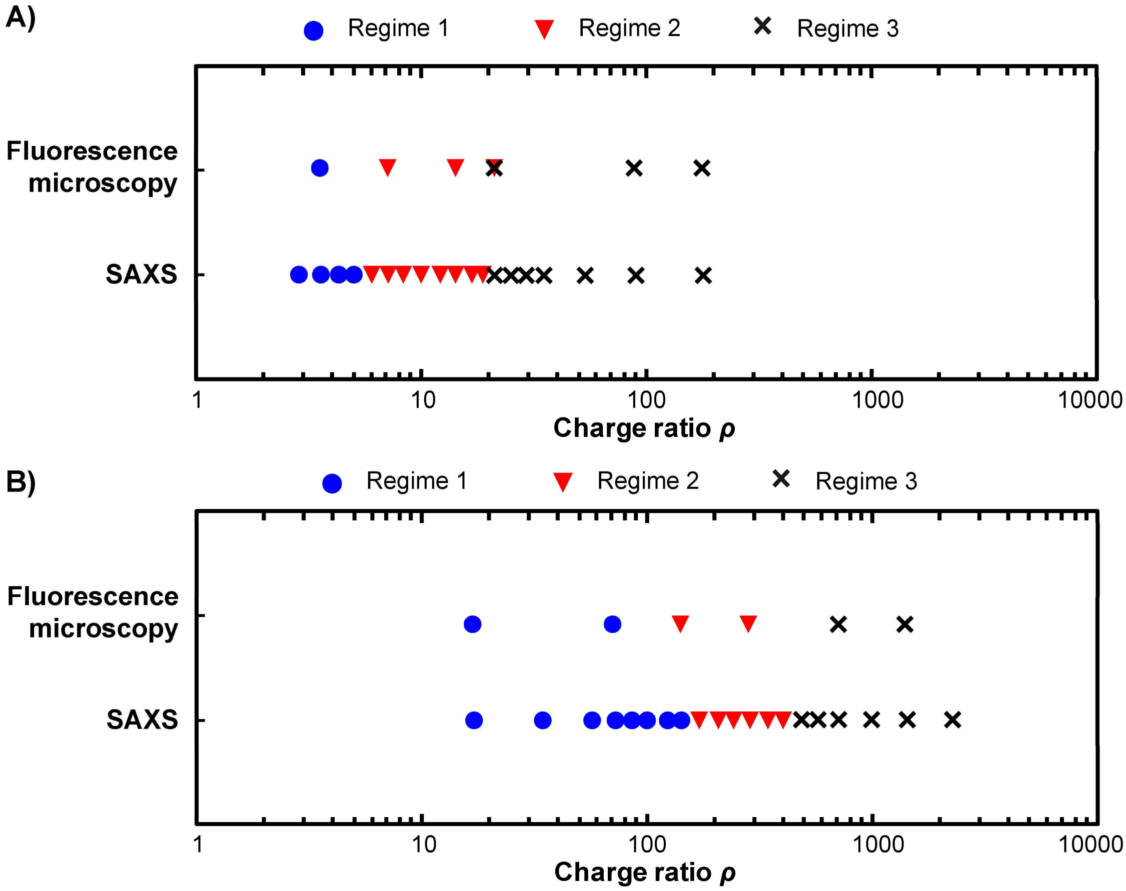

Figure S12. Diagrams showing the types of regimes of nanotube assembly determined by fluorescence microscopy (top) and SAXS (bottom) as a function of the charge ratio, upon addition of A) SPM^{4+} and B) SPD^{3+}. Each DNA strand concentration is 500 nM in TAMg buffer.

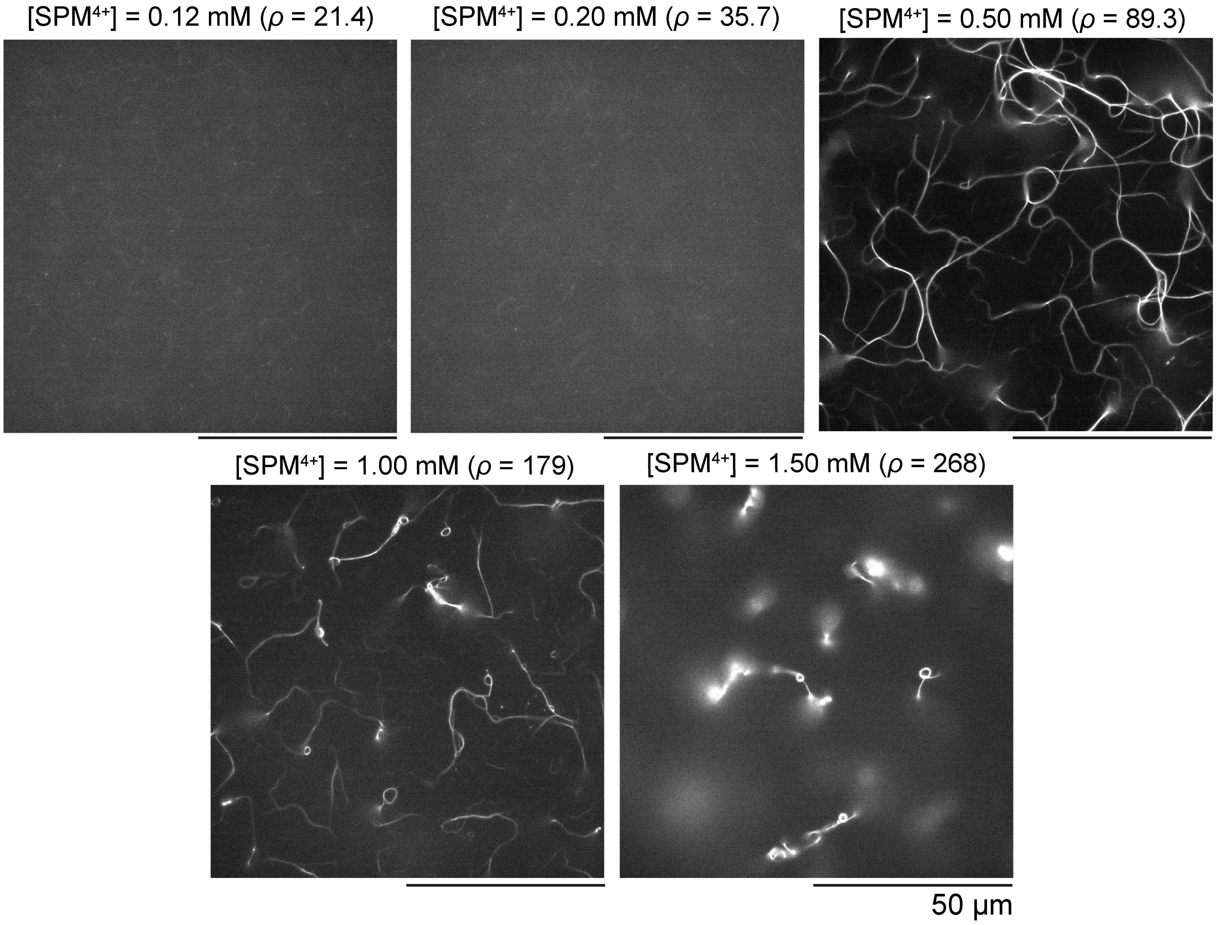

Figure S13. Epifluorescence images of DNA structures and superstructures obtained after the addition of different amount of spermine (SPM^{4+}) to individual DNA nanotubes. Each DNA strand concentration is 100 nM in TAMg buffer.

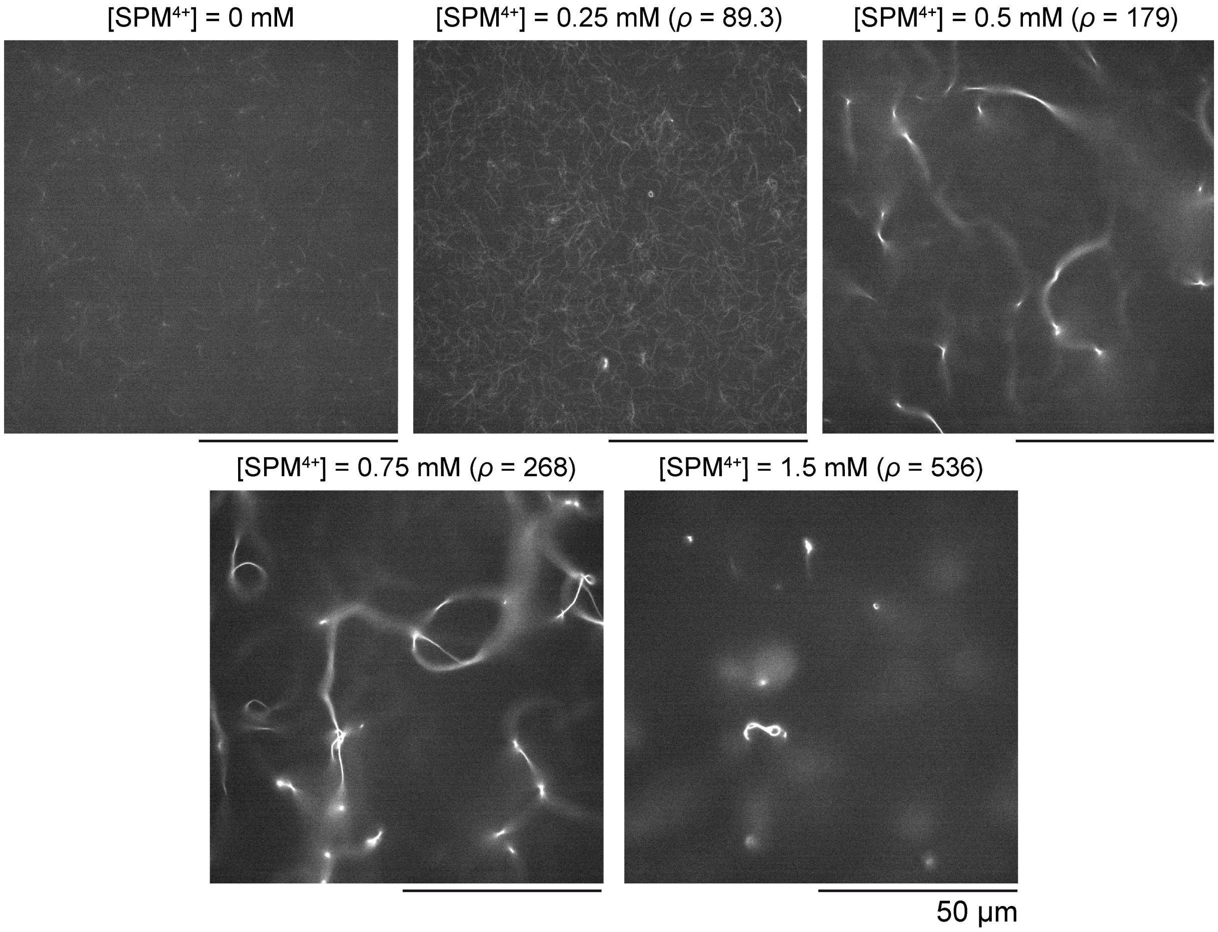

Figure S14. Epifluorescence images of DNA structures and superstructures obtained after the addition of different amount of spermine (SPM^{4+}) to individual DNA nanotubes. Each DNA strand concentration is 50 nM in TAMg buffer.

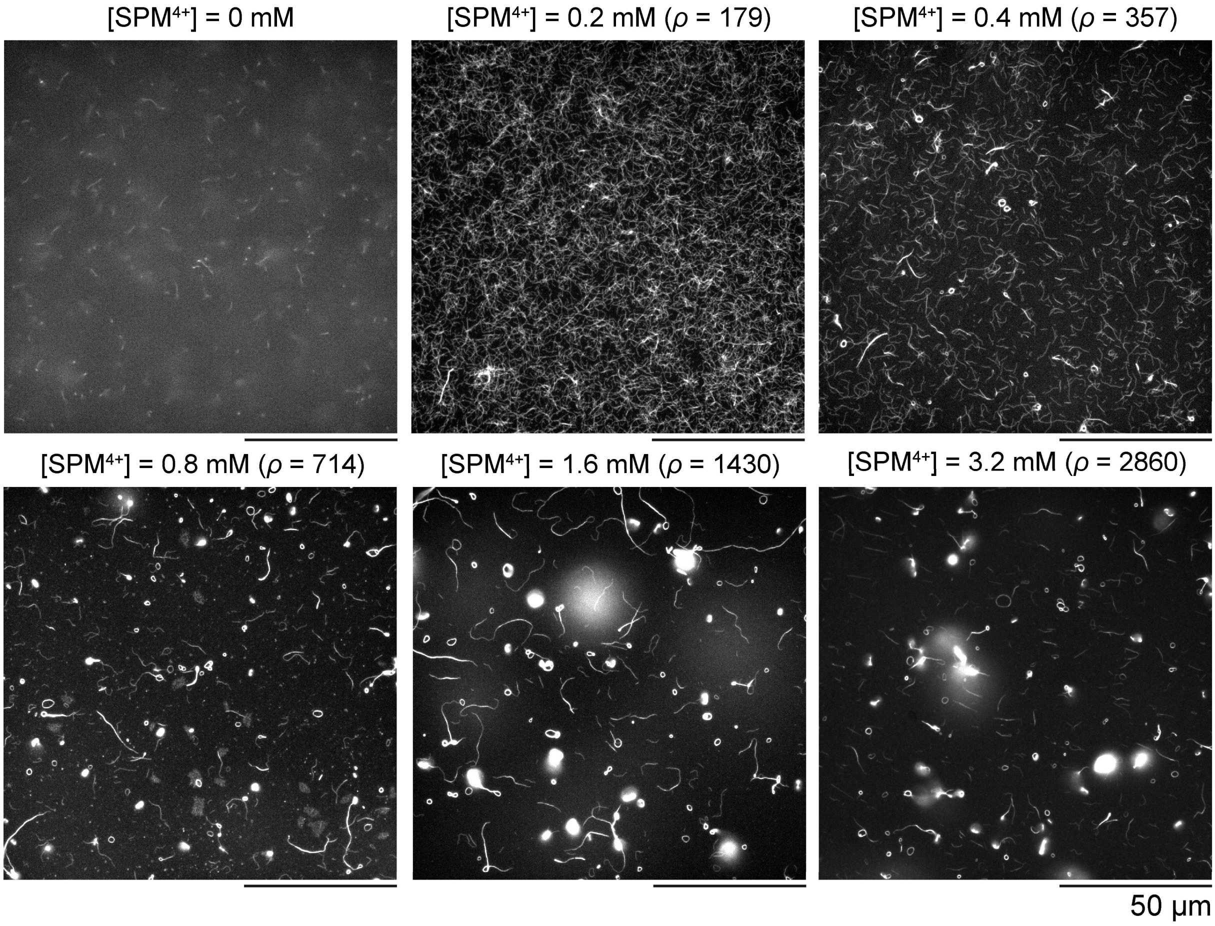

Figure S15. Epifluorescence images of adsorbed DNA structures and superstructures obtained after the addition of different amount of spermine (SPM^{4+}) to individual DNA nanotubes. Each DNA strand concentration is 20 nM in TAMg buffer.

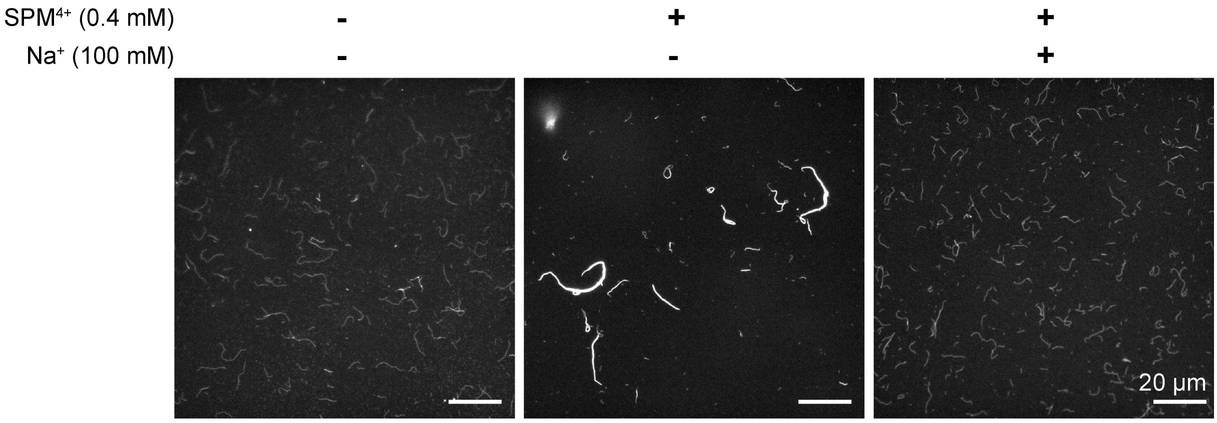

Figure S16. Representative epifluorescence microscopy images of adsorbed individual nanotubes or DNA superstructures obtained after the addition of 0.4 mM spermine, and the consecutive addition of 0.4 mM spermine and 100 mM NaCl. Each DNA strand concentration was 500 nM in TAMg buffer and diluted to 5 nM for observation.

4. Legends of the Supplementary Movies

Movie S1. Epifluorescence microscopy observation of the formation of DNA networks or bundles induced by the condensation of DNA nanotubes with the addition of 0.4 mM or 2.5 mM of spermine, respectively. The movie is displayed at real time.

Movie S2. Epifluorescence microscopy observation of the reversible formation and dissociation of DNA networks induced by the addition of 0.4 mM of spermine and the further addition of 100 mM NaCl. The movie is displayed at real time.

Movie S3. Epifluorescence microscopy observation of the photosensitive formation of DNA networks induced by the addition of AzoTAB. The movie is displayed at real time.

5. Supplementary references

(1) Bencini, A.; Bianchi, A.; Garcia-España, E.; Micheloni, M.; Ramirez, J. A. Proton Coordination by Polyamine Compounds in Aqueous Solution. *Coord Chem Rev* 1999, *188* (1), 97–156. https://doi.org/10.1016/S0010-8545(98)00243-4.

(2) Weisell, J.; Hyvönen, M. T.; Vepsäläinen, J.; Alhonen, L.; Keinänen, T. A.; Khomutov, A. R.; Soininen, P. Novel Isosteric Charge-Deficient Spermine Analogue—1,12-Diamino-3,6,9-Triazadodecane: Synthesis, PK a Measurement and Biological Activity. *Amino Acids* 2010, *38* (2), 501–507. https://doi.org/10.1007/s00726-009-0409-6.
